# Integrating fuzzy AHP and geo-spatial modeling for wind farm suitability assessment in Kuwait

**DOI:** 10.1038/s41598-026-46695-4

**Published:** 2026-04-03

**Authors:** Mohamed A. Atalla, Ayad M. Fadhil Al-Quraishi, Elsayed A. Badawy Ataalla, Ali Shebl, Árpád Csámer, Wael M. Al-Metwaly

**Affiliations:** 1 Department of Natural Studies, General Organization for Physical Planning (GOPP), Ministries District, New Administrative Capital, Cairo, 85863 Egypt; 2 Department of GIS and Environment, Enshaat Al-Sayer General Trading and Contracting Company, Floors 14 & 19, Panasonic Tower, Safat, P.O. Box 126, Kuwait City, 13002 Kuwait; 3 Petroleum and Mining Engineering Department, Tishk International University, Erbil, Iraq; 4 Architecture Engineering Department, Faculty of Engineering, Tanta University, Tanta, 31527 Egypt; 5 Construction Department, Lamsat Al Zahra Building Contracting L.L.C, Office No. 124-103, Al Murar, Deira, Plot No. 117-678, Dubai, United Arab Emirates; 6 Department of Geology, Tanta University, Tanta, 31527 Egypt; 7 Department of Mineralogy and Geology, University of Debrecen, Debrecen, 4032 Hungary; 8 Cosmochemistry and Cosmic Methods Research Group, University of Debrecen, Debrecen, 4032 Hungary; 9 Department of Geography and GIS, Faculty of African Postgraduate Studies, Cairo University, Giza, 12613 Egypt

**Keywords:** Wind farm sitting, Sustainable development goals (SDGs), GIS, Fuzzy AHP, Multi-criteria decision making (MCDM), Geo-spatial modeling, Kuwait, Energy science and technology, Engineering, Environmental sciences, Environmental social sciences

## Abstract

In response to global energy depletion, this study addresses the critical challenge of selecting optimal wind farm sites in Kuwait, a key pillar of the “New Kuwait” 2035 vision for 15% renewable energy integration. A robust, hybrid Multi-Criteria Decision-Making (MCDM) framework was developed, integrating Fuzzy Analytical Hierarchy Process (FAHP) with Entropy-based objective weighting and Type-2 Fuzzy logic within a GIS environment. This approach effectively mitigates expert subjectivity and manages the high “Footprint of Uncertainty” inherent in complex desert environments. Twenty-six technical, environmental, and socioeconomic criteria—including wind power density, proximity to the national grid, and oil field restrictions—were analyzed at a modern utility-scale hub height of 100 m. The spatial results identified approximately 1444 km² (8.6%) of Kuwait’s territory as ' Highly Suitable’ for wind development, primarily concentrated in the western (Al-Jahra) and northern (Al-Abali) corridors. Validation against existing Shagaya Renewable Energy Park and Global Wind Atlas data confirmed a strong spatial correlation (R^2^ = 0.84). The identified zones represent a potential generation capacity of 2500 MW, providing a data-driven roadmap for national energy planners to meet sustainability targets while minimizing land-use conflicts with oil production and urban expansion.

**Supplementary Information:**

The online version contains supplementary material available at 10.1038/s41598-026-46695-4.

## Introduction

Electricity generation from renewable sources is pivotal in driving social and economic development, especially given the global shortage of fossil fuels, such as oil and natural gas. Moreover, numerous countries have adopted policies prioritizing renewable energy sources to combat global warming and reduce greenhouse gas emissions^1–3^. Consequently, extensive research has been conducted to explore and harness renewable energy sources, particularly solar and wind power, owing to their crucial roles in promoting energy independence and mitigating the adverse effects of climate change^4–6^. Identifying suitable locations for establishing renewable energy power plants is an essential initial phase in the sustainable advancement of renewable energy sources^7^. This task poses a complex challenge for energy planners and policymakers and requires careful consideration of a diverse array of competing factors^8^. The Multi-Criteria Decision Making (MCDM) methodology is a viable approach for thoroughly assessing and prioritizing alternatives from diverse viewpoints^2^. Numerous researchers have developed and applied different MCDM techniques to identify optimal sites for wind and solar photovoltaic power plants^5,9^. Various methodologies have been developed to address these issues.

These approaches incorporate a wide spectrum of MCDM techniques, such as: the fuzzy analytic hierarchy process (FAHP), technique for order of preference by similarity to ideal solution (TOPSIS) and its fuzzy extension (F-TOPSIS), data envelopment analysis (DEA), weighted aggregated sum product assessment (WASPAS), and stepwise weight assessment ratio analysis (SWARA). By employing these methods, the decision-making process can be enhanced, and optimal outcomes can be attained^10^.

Nonetheless, identifying appropriate locations for installing renewable energy sources poses a formidable challenge owing to the diverse data involved and the conflicting criteria that must be considered throughout the selection process^9,11^. To overcome these challenges, integrating MCDM techniques with GIS has emerged as a widely adopted framework for renewable energy site selection. This synergy enhances spatial decision-making by systematically incorporating multiple, and often conflicting, criteria within a comprehensive geospatial environment^12^. Numerous studies have used GIS and GIS-MCE (Multi-criteria Evaluation) approaches to identify suitable sites for wind farm development. For instance, Van Haaren et al.^13^ used GIS to determine the optimal location for a wind farm in New York, United States. Similarly, Tegou et al.^14^ used the GIS-AHP method to locate wind farms on the island of Lesvos in Greece. Similarly, Georgiou et al.^15^ utilized the GIS-AHP method to identify suitable areas for a wind farm in the Larnaca province of Cyprus. Their findings indicated that only a small fraction (0.1% of the area) displayed high potential for wind turbine construction.

In contrast, Aydin et al.^16^ employed the GIS-MCDM method to identify the optimal location for a wind farm in western Turkey. Their study highlighted environmental factors as the most significant drivers of water quality in the study area. Another study by Albraheem et al.^17^ focused on the location of wind farms in Saudi Arabia. Initially, they generated wind GIS maps using an interpolation technique, followed by the GIS-AHP method to identify suitable regions. Notably, Ras Tanura, Turaif, and Al-Wajh emerged as the most suitable areas for wind farm development. Höfer et al.^18^ conducted a comprehensive study to determine the optimal locations for a wind farm in the Städteregion Aachen, Germany.

In Kuwait, Al-Nassar et al.^19^ provided a seminal assessment of wind power potential by analyzing data from six inland and coastal stations. Their findings, based on Weibull distribution parameters, demonstrated that wind power density significantly increased with altitude (a 70% increase from 10 to 30 m) and identified the northern regions as having the highest generation potential. Although their work established the meteorological feasibility of wind energy in Kuwait, it was constrained by the low hub heights (30 m) at the time and a limited number of observation points. Our study builds upon this foundation by extending the analysis to a 100-m hub height and employing a high-resolution GIS framework to provide continuous spatial suitability across the entire country.

In recent years, extensive research has identified optimal locations for renewable energy facilities, and wind power has made significant advancements, achieved notable strides, and demonstrated substantial progress. Over the past 15 years, the installation of wind energy systems has consistently increased by 9% per year^20^. The adoption of wind energy is increasing in both developed and developing nations, indicating its growing popularity. A notable example is Denmark, where wind energy accounts for 40% of household energy consumption^21^. Furthermore, China has experienced a substantial surge in its wind energy generation capacity, soaring from 19.3 GW in 2016 to 149 GW recently^22^. Saudi Arabia has embarked on a journey to develop wind farms. A significant milestone has been set with the ongoing establishment of a 400 MWh wind farm in Dumat AlJandal^21^. These examples demonstrate how wind energy offers a sustainable alternative to fossil fuels, inspiring and encouraging other countries to adopt this clean, renewable energy source.

Recent literature (2022–2025) reflects a significant shift toward integrating Geographic Information Systems (GIS) with advanced uncertainty-handling techniques and hybrid Multi-Criteria Decision-Making (MCDM) frameworks. For instance, Hoang et al. ^23^ and Placide and Lollchund^24^ demonstrated the efficacy of coupling GIS with Fuzzy Logic relations and mathematical modeling to refine site selection in Vietnam and Burundi, respectively. Several studies have emphasized the role of hybrid models in mitigating subjectivity; Nguyen et al. ^25^ introduced a spherical fuzzy MCDM model for turbine selection, while Zeynep^26^ and Aghapour et al.^27^ utilized cluster-based hybrid machine learning and MCDM approaches to optimize site identification in Turkey and Iran, respectively.

The geographical breadth of these applications is evident in recent regional studies. In Turkey, Yaman^28^ and Gülay Demir et al.^29^ applied GIS-based MCDM and fuzzy decision-making to identify onshore wind potential, while Demir et al.^30^ further advanced the field by integrating life cycle assessment (LCA) into spatial multicriteria analysis. Similarly, extensive research in Iran by Alavi et al.^31^, Yousefi et al.^32^, and Razeghi et al.^33^ has highlighted the versatility of MCDM in diverse contexts, including the use of wind energy to power reverse osmosis desalination systems.

Furthermore, the focus has expanded to address specific operational and strategic challenges. Dhingra et al.^34^ and Richards et al.^35^ explored the prioritization of barriers and stakeholder perspectives to ensure sustainable onshore and offshore planning. In low-wind regimes, Andi Luku et al.^36^ and Malka et al.^37^ investigated the strategic selection of turbines and the associated environmental benefits. Ozer Eroğlu et al.^38^ provided a comprehensive synthesis of the literature, confirming that the synergy between GIS and hybrid MCDM remains the state-of-the-art approach for robust renewable energy infrastructure planning.

Finally, a recent study by Hasan et al.^39^ explored the potential of offshore wind energy in Kuwait’s territorial waters using a GIS–FAHPP framework. Although their research highlights the vast marine wind resources, offshore projects involve significantly higher capital expenditures (CAPEX) and complex maritime logistics than onshore developments. Therefore, our study fills a critical gap by providing a high-resolution, multi-criteria assessment of onshore wind suitability. By focusing on the desert interior, which is more accessible for immediate infrastructure integration, this research offers a more cost-effective, logistically feasible roadmap for Kuwait’s short- to medium-term renewable energy targets.

This study builds upon these recent advancements by incorporating a hybrid FAHP-Entropy-Type- 2 Fuzzy framework, which offers greater precision in uncertainty management than the methods described in earlier literature, particularly crucial for Arab Gulf nations striving to promote renewable energy sources in line with European standards. However, in many cases, broad political objectives have been formulated without an initial assessment of a region’s actual potential. Compliance requirements and associated limitations should be addressed. Different studies have established criteria for compatibility but have failed to clarify their sources. Some studies have adopted the authors’ standards across various regional contexts. It is essential to recognize that such approaches can yield inaccurate results because the criteria and thresholds must align directly with the specific characteristics of each site. Therefore, caution must be exercised when applying standards developed in other fields, as they require evaluation by experts in the respective research fields and by professionals knowledgeable about the specific subject area.

We implemented the most stringent suitability criteria based on the existing literature. However, it is essential to note that the results regarding the suitability of Kuwait’s land for wind farm installation are valid only at a general level of analysis. Subsequent comprehensive assessments should be conducted by incorporating additional suitability standards at the sub-regional or local levels. These broader evaluations can be valuable tools for supporting planners and investors in their decision-making. The findings of our investigation indicate that Kuwait is a suitable location for such studies. However, it is essential to note that these conclusions are broad and must align with the analysis’s scope. For accurate wind resource assessments, extensive on-site testing lasting at least one year, followed by meticulous data analysis, is necessary.

The findings revealed that Kuwait has significant solar and wind potential. These results compare favorably with those of other studies and serve as motivating factors for investors and policymakers. The Kuwaiti government has set an ambitious target to achieve 30% electricity generation from renewable sources by 2030. This objective aims to ensure energy independence, diversify the energy mix, and reduce emission intensity. Interestingly, the identified potential areas for renewable energy development are located in the country’s least developed western regions. To include these regions in the development process, concrete actions must be taken to attract investment in solar and wind projects. This includes establishing manufacturing facilities, educational and training institutions, and enhancing the grid and transportation infrastructure. These efforts will contribute to the optimal placement of renewable energy facilities, positively impacting social welfare and overall living conditions. Kuwait aims to diversify its energy sources and increase the share of renewable energy in its energy mix as part of its Vision 2035 strategy. The focus on wind energy aligns with global trends toward sustainability and energy independence.

### Research gap and its aim

Despite global advancements in wind energy mapping, studies in the Arabian Gulf, and particularly in Kuwait, often suffer from three primary limitations:

1. *Methodological Staticity*: Most previous assessments relied on traditional AHP, which fails to account for the inherent subjectivity and uncertainty in expert judgments.
2. *Criteria Narrowness*: Existing literature often focuses on primary wind speeds and overlooks the complex interplay of socioeconomic and environmental constraints specific to Kuwait’s landscape (e.g., oil-field buffers and migratory pathways).
3. *Uncertainty Management*: There is a notable lack of integration between objective data-driven weighting (entropy) and subjective expert modeling (type-2 fuzzy) in the regional context.

This study addresses these gaps by developing a hybrid MCDM framework that integrates FAHP, entropy-based weighting, and Type-2 FL within a GIS environment. This approach enhances the precision of site selection and provides a dynamic, uncertainty-aware model that aligns with Kuwait’s strategic 2035 energy goals.

This GIS-based MCDM-FAHP enables geoprocessing, analysis, and conversion of geographical/spatial and non-spatial data into valuable information, which, when combined with decision-makers’ judgment, facilitates critical decision-making processes^40,41^.

This study aims to address Kuwait’s significant reliance on fossil fuels in the energy sector. Kuwait is among the top countries in terms of water and electricity consumption, consuming over 350,000 barrels of fuel daily, which amounts to more than 2 billion Kuwaiti dinars annually. The high demand for electricity and water in Kuwait is driven by its hot, arid climate and rapid economic growth. Additionally, Kuwait ranks seventh globally in annual per capita electricity consumption, with an average of 14,900 kWh. Despite the presence of power plants with a total installed capacity of 15,000 MW, production is expected to reach 32,000 GWh by 2030. Kuwait has established solar energy facilities as part of its renewable energy initiatives. Notable projects include the Kuwait Institute for Scientific Research’s (KISR) operational station, which is designed to produce 100 kW of thermal power. The Al-Shagaya project aimed to reach a capacity of 4,000 MW, supplying the national grid with 23 million kWh over six months. Other significant projects, such as Al-Dabdaba, Sidra, and Al-Abraq, have electricity capacities of 1,500 MW, 500 MW, and 1,700 MW, respectively. These initiatives have contributed to meeting 15% of domestic electricity demand, saving 12.5 million barrels of fuel annually, and reducing carbon emissions by approximately 5 million metric tons^3^.

Kuwait’s wind energy production has notably increased in recent years, reflecting the country’s strategic shift towards renewable energy sources (Table 1). From the initiation of wind projects in 2017, when production stood at 10,000 MWh, to a substantial 35,000 MWh in 2023 and 50,000 MWh by 2025, this upward trend highlights the effectiveness of efforts to harness wind energy. Key areas, such as Al-Jahra and Bar Al-Jahra, have emerged as significant hubs for wind energy generation. The continuous development and operationalization of new wind farms underscore the commitment to diversifying the energy mix and addressing the growing energy demand in Kuwait^42–44^.

**Table 1:** Annual wind energy production in Kuwait (2017–2025).

Year	Amount generated (MWh)	Highest production area	Notes	Source
2017	10,000	Al-Jahra Governorate	Start of wind energy projects	KISR
2018	15,000	Al-Ahmadi Governorate	Increased wind capacity	Kuwait Ministry of Electricity and Water (MEW)
2019	20,000	Bar Al-Jahra	New wind farms operational	Kuwait National Petroleum Company (NPC)
2021	25,000	Al-Jahra Governorate	Initial wind energy projects launched	KISR
2022	30,000	Al-Salmi	Significant growth in wind capacity	MEW
2023	35,000	Bar Al-Jahra	Ongoing development of wind projects	NPC
2024	42,000*	Shagaya (Al-Jahra)*	Expansion of Shagaya Phase II	KISR/MEW (2024)*
2025	50,000*	Shagaya (Al-Jahra)*	Integration of advanced turbines	KISR/MEW (2025)*

## Materials and methods

Figure 4 illustrates the methodological steps followed in this study, which are detailed as follows:

### The study area

**Fig. 1:**
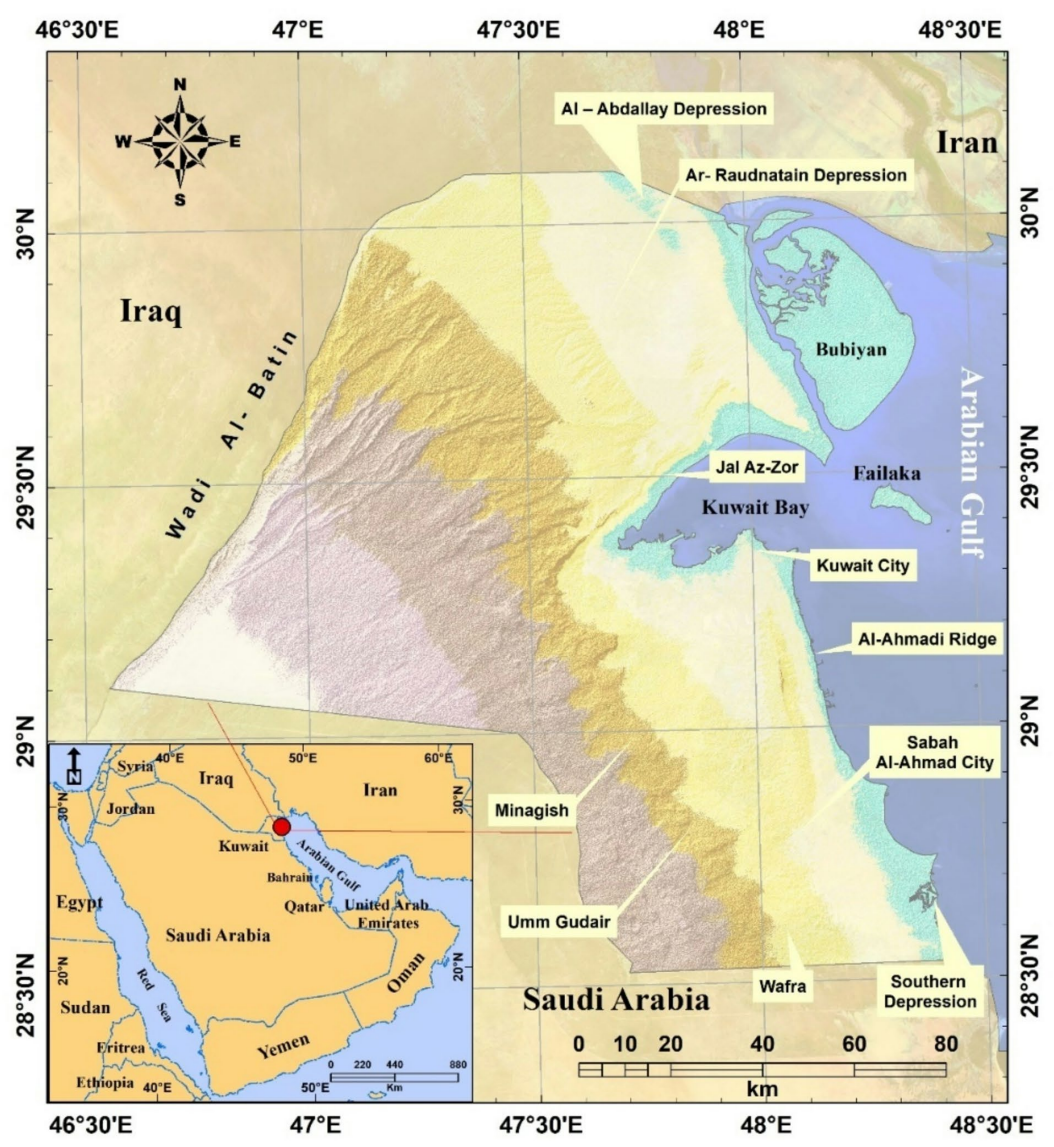
Location map of Kuwait.

The entire area of Kuwait was included in this study, which lies to the northwest of the Arabian Gulf between latitudes 28 °30 ' and 30 °05 ' N and longitudes 46 °33 ' and 48 °30 ' E. Figure 1 illustrates that Kuwait shares borders with the Arabian Gulf to the east (483.8 km), Iraq to the north and west, and Saudi Arabia to the south and west. Kuwait’s total land area is 18,000 km^2^, and most of its population (4.67 million) is concentrated in the Arabian Gulf. Kuwait has a hot, arid climate, characterized by high summer temperatures. The summer temperature ranges from 42 to 46°C^45,46^. Occasional rainfall occurs in spring, with an annual average of 75–150 mm. Dust storms occasionally occur in the summer. The annual average relative humidity is 60%, but it can reach 84% during thunderstorms. The daily evaporation rate in Kuwait was approximately 10.9 mm.

**Fig. 2:**
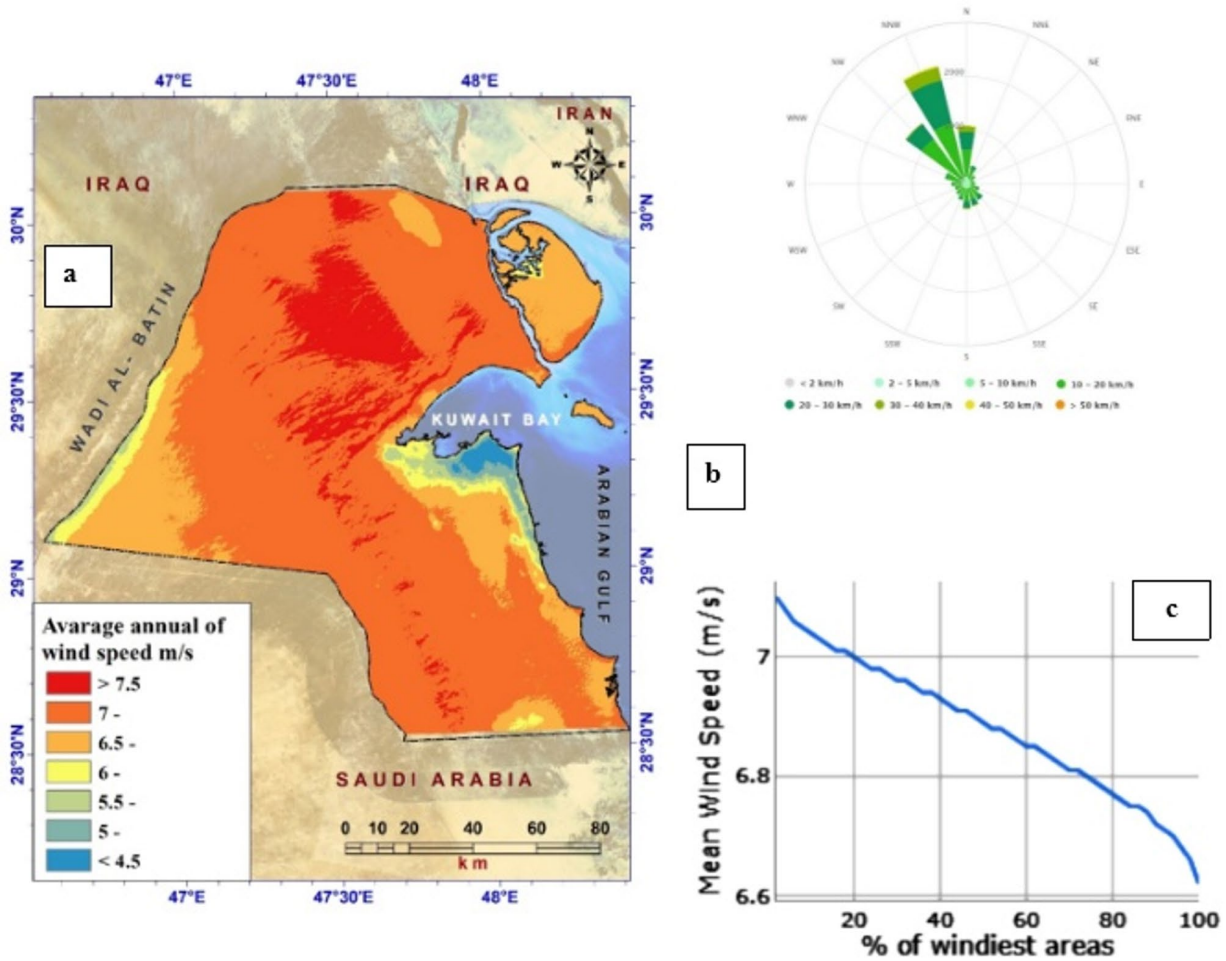
Wind characteristics of Kuwait. **a** Average annual wind speed at 50 m height; **b** wind rose diagram; **c** Mean Wind Speed at Height of 50 m.

Regarding wind patterns, Kuwait experiences wind speeds ranging from 3.5 m/s to 7.79 m/s, with a prevailing wind direction from northwest to southeast. It is worth highlighting that wind speeds tend to increase as one moves toward the central and western parts of the country. This presents a promising opportunity to harness wind energy generation, as shown in Fig. 2.

**Fig. 3:**
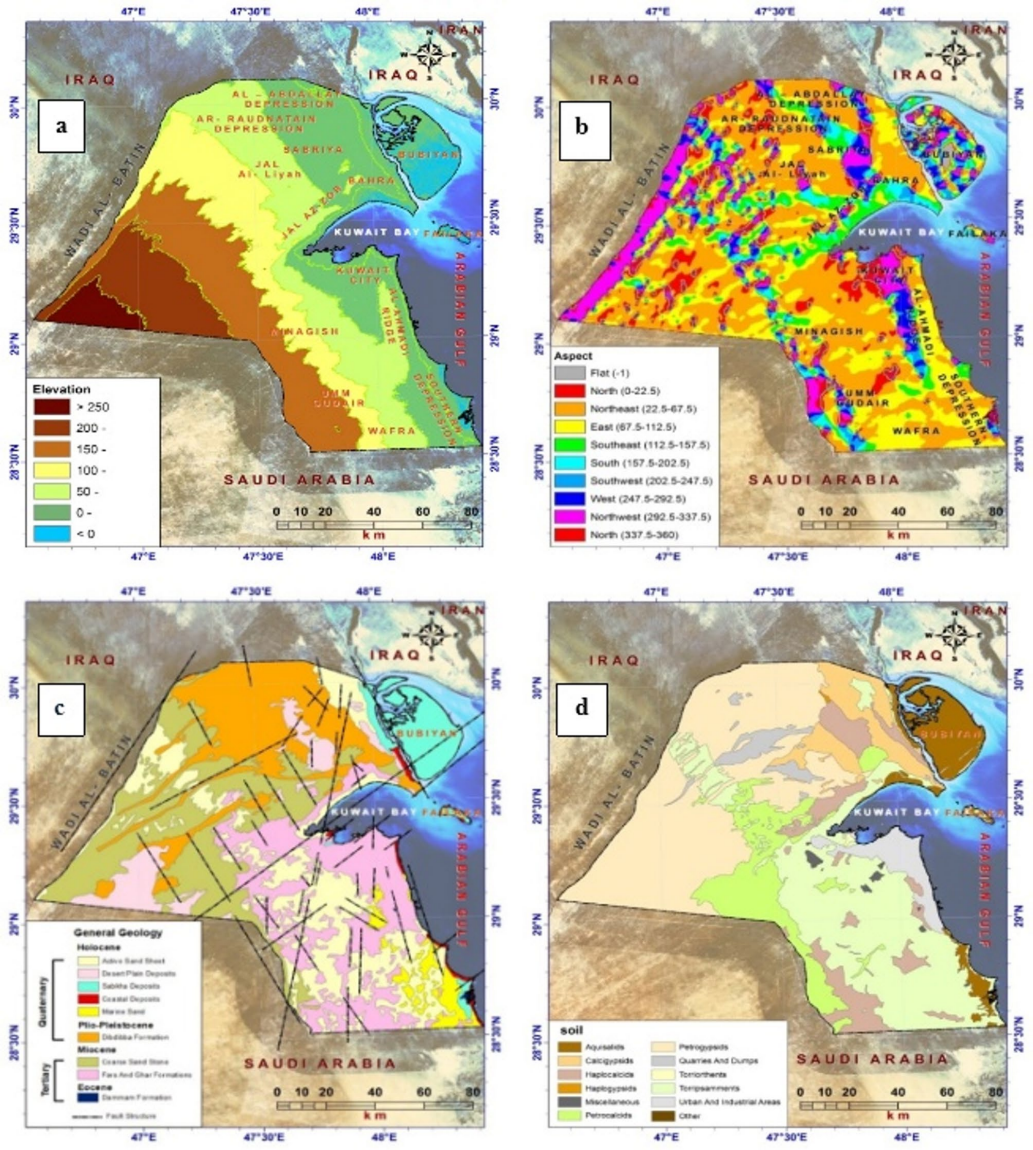
General geographical attributes of Kuwait. **a** Topographic map; **b** slope directions; **c** geological map; **d** soil type map.

The topography of Kuwait is characterized by low local relief, which gradually increases from the shores of the Arabian Gulf towards the southwest (288 m.a.s.l.), except for some hills and cliffs along the northern coast of the Gulf of Kuwait, such as the Jal al-Zour cliff and Al-Ahmadi hills parallel to the east coast of Kuwait. Some small hills are also found along the western borders of Wara, Burgan, and Wadi Al-Batin (Fig. 3a). Most slope grades were less than 5° to the east and northeast of the study area (Fig. 3b). Notably, the southeastern part of Kuwait is characterized by silt, marshes, and ponds that are below sea level. The northeastern part of Kuwait has low-lying clay beaches and several oceanic islands, with Boubyan being the largest. The area is believed to be the site of delta deposits and estuaries in the Tigris River System. None of the islands on the surface is alluvial or muddy, though some windblown sand reaches heights of 10 feet or more. Large areas of clay surround the islands.

Kuwait’s surface geology is still under debate; however, it is composed of four geological ages belonging to the Tertiary and Quaternary periods, with the oldest being the Eocene limestone, referred to as the Dammam Formation, followed by the Miocene Ghar, Lower Fars, and Dibdibba formations. The Holocene epoch represents the most recent formation and is characterized by dunes, alluvial and desert sediments, marshes, and coastal and marine sediments (Fig. 3c). Most deposits in Kuwait have been composed of sand, silt, and marine, coastal, and desert sediments since the Holocene. The Pleistocene and Miocene periods consist of coarse sand, clay chips, gravel, sand, and limestone. The soil in Kuwait is characterized by low organic matter content and a high percentage of salt and calcareous materials, due to its submersion beneath the sea surface in ancient geological times and the subsequent recession of water in the Quaternary^46,47^ (see Fig. 3d).

### Datasets

Various datasets were combined to accomplish the study goals, as shown in Table 2. One of the key datasets used was the Shuttle Radar Topography Mission digital elevation model (DEM), which has a spatial resolution of 30 m × 30 m. This dataset was crucial for extracting the topographical and geomorphological details necessary for analysis^48^, including slope and aspect. Additionally, various remote sensing maps and the Environmental Monitoring Administration Information System for the State of Kuwait were integrated to enhance data comprehensiveness. The statistical population data for Kuwait in 2019 were obtained from the Central Administration for Statistics. Furthermore, the Global Wind Atlas, climate data, Sentinel-2-based land use and land cover (LULC) data for Kuwait in 2020, and OpenStreetMap’s (OSM) free wiki world map data were incorporated. Several software tools were employed to process these datasets, including ArcGIS (Ver. 10.8), ERDAS Imagine (2022), Surfer (ver. 17, Golden Software Inc.), and Global Mapper (Ver. 19). Georeferencing was applied to align all maps with the borders of the study area.

### Methodology

**Fig. 4:**
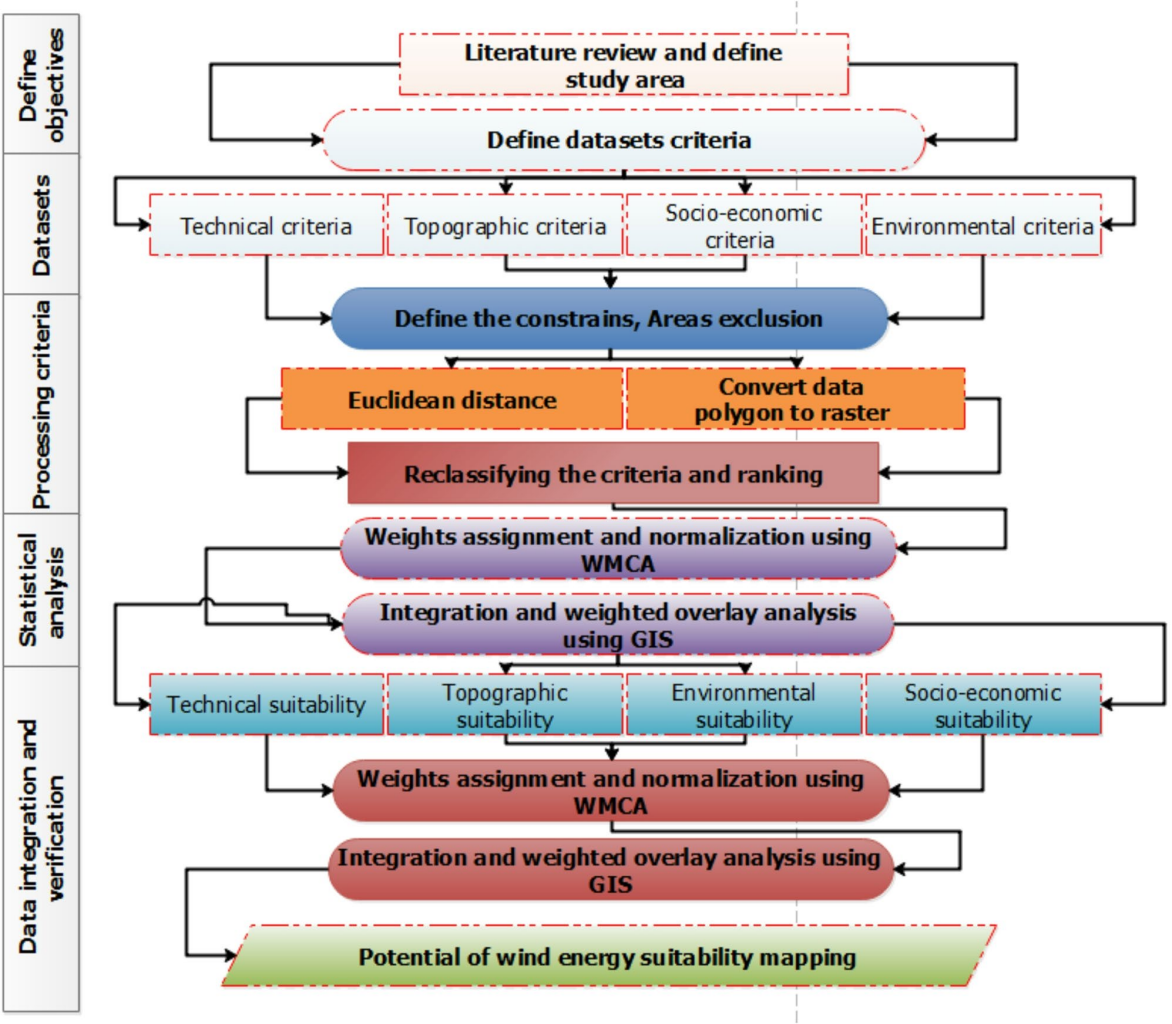
Methodological flowchart of the weighted multi-criteria analysis process adopted in this study.

As shown in Fig. 4, this study employed multiple criteria and thematic layers as part of a suitability analysis to map wind energy potential. These layers had varying levels of importance, determined through a weighted overlay analysis. Each layer was assigned a numerical weight based on its impact on the final output, yielding a quantitative assessment of how well-suited a particular location (or “pixel”) was for mapping the desired feature. Using GIS as a flexible and powerful tool for entering, analyzing, and integrating various data sources, a comprehensive digital database for the study area was created, as illustrated in Fig. 4 to demonstrate the methodology used. Initially, a geodatabase was created in ArcGIS, and spatial and hydrological analyses were used to extract topographical data, including terrain, valleys, and water drainage basins and networks.

A multi-criteria analysis (MCA) method was employed to assess the suitability of specific areas for various applications. This method was developed to enhance spatial decision-making when evaluating a set of alternatives using conflicting, equally weighted criteria, represented by a combination of qualitative and quantitative maps. The spatial modeling process using MCA combines data and information that represent the criteria into a single form to build a new indicator that determines the suitability of a particular site for specific purposes. This indicator assesses the relative importance of each criterion using a logical evaluation process. Based on the GIS analysis data, we applied a multi-criteria modeling approach using these data as input variables.

The selection of suitable sites for power plants in Kuwait was achieved through six steps, as shown in Fig. 4: (1) setting up the database, (2) setting the appropriate criteria for site selection, (3) addressing the criteria, (4) reclassifying the criteria, (5) weighting the acceptable regions and their importance, and (6) applying the model considering the relevant social and economic factors to assess the suitability of specific areas for optimal positioning. Some of these steps were implemented using spatial analysis and the model builder in GIS to produce appropriate maps that suggest the “most appropriate” geographic regions derived from the balanced and grouped map layers based on the criteria defined in Phases I and II, as follows:

The optimal site selection process involves assessing multiple factors represented by a set of factor maps, whereas constraints are visualized through constraint maps during database construction. This implies that the various criteria, initially presented as vectors, were converted to raster format. This transformation enabled a more comprehensive and spatially explicit analysis of the criteria and constraints of site selection. These criteria have been examined using various approaches discussed in previous studies, which have contributed to shaping planning considerations. The analysis of potential sites for wind farm establishment focused on several factors, which can be categorized into four groups (Table 2): topographic, technical, environmental, social, and economic^49^.

The threshold values and buffer distances adopted in this study were specifically tailored to reflect Kuwait’s unique regulatory, environmental, and sociopolitical landscapes.

1. Regulatory Context: The values align with the executive regulations of the Kuwait Environment Public Authority (EPA) and the Kuwait Municipality’s master plan, ensuring that buffer zones around residential areas and nature reserves meet national safety and zoning standards.
2. Environmental Context: Given Kuwait’s arid climate and fragile coastal ecosystems, the thresholds for the coastline and protected areas were adjusted to account for the high sensitivity of sabkhas and desert habitats to anthropogenic disturbances. The threshold values used in this study were refined in accordance with the guidelines of the EPA and the Fourth Kuwait Master Plan 2040, thereby ensuring that the model respects the scarcity of land and the fragility of arid ecosystems.
3. Socio-political context: This selection reflects Kuwait’s urban expansion policies and industrial zoning strategies, which aim to balance rapid development with environmental conservation, as outlined in the Kuwait National Development Plan (Vision 2035).

The selection of the main criteria and their corresponding sub-criteria followed a dual-phase approach. First, a comprehensive review of the existing literature on wind farm siting in arid regions was conducted to identify the most influential factors. Second, these factors were refined and categorized into three main clusters (topographic, technical, and environmental) to suit Kuwait’s specific geographic conditions and data availability. Table 2 summarizes these factors, along with their definitions and rationale for inclusion.

**Table 2:** Factors used in the analysis of wind farm suitability.

Factors	Criteria	Source	Datasets
Topographic			
Elevation	< 1500 m A.S.L.	^49^	DEM, 2019 (https://earthexplorer.usgs.gov/)
Slopes	< 10°	^50,51^	
Aspects	315°< Value < 360° (NW - N)	^46^	
	0 < Value < 45 (N - NE)		
Vacant area	> 100.000 m^2^ (10 ha)	^52^	LU/LC map, 2024 (https://livingatlas.arcgis.com/landcover/)
Geology	Avoidance of sabkhas, valleys, and coastal sediments	NA	Geological formations map ^33^ (https://beatona.net/en/apps)
Soils	Avoidance of loose soil from limestone and water deposits		Soil types of map (https://beatona.net/en/apps)
Technical			
Wind speed	> 5 m/sec	^23,24^	Average wind speed (1979–2025); From: Technical University of Denmark DTU Global Wind Atlas 1 Km Resolution (https://globalwindatlas.info/en/); and (https://power.larc.nasa.gov/)
Proximity to the power grid	500 m < value < 1000 m		Infrastructure map (https://www.google.com/maps/)
Environmental			
Proximity to shoreline	> 500 m	^28,29^	Shoreline (Contour 0) extracted from DEM (https://earthexplorer.usgs.gov/)
Sand movement	Avoidance of high sand movement areas	NA	Applying change detection using normalized differences sand index (NDSI) on multi-temporal landsat imagery
Land cover	> 500 m of woodland & wetland areas & water bodies	^53^	LU/LC map, 2024 (https://livingatlas.arcgis.com/landcover/)
Proximity to protected areas	> 500 m	^31–33^	
		^53–55^	
Proximity to drainage network density	Avoidance of high drainage network density		Applying the hydrology model on DEM and using the drainage density tool from the ArcGIS toolbox (https://earthexplorer.usgs.gov/)
Proximity to faults	Avoidance of high fault density		Geological map (Structure) (https://www.google.com/maps/)
Proximity to seismic hazard	Avoidance of high seismic hazards		Seismic hazards map^36^
Proximity to seismicity distribution	Avoidance of high seismicity distribution density		Seismicity distribution map^37^
Other factors			
Proximity to an urban area (city)	> 2000 m	^50^	Topographic map (https://www.mapsland.com/maps/asia/kuwait/large-topographical-map-of-kuwait.jpg)
Proximity to military areas	> 500 m		
Historical sites	> 1000 m		
Proximity to the industrial area	> 500 m		
Proximity to roads	250 m < Value < 10,000 m	^49^	Infrastructure map (https://www.google.com/maps/)
Proximity to airports	> 3000 m		
Proximity to railways	250 m < Value < 10,000 m		
Buffer distance of oil and gas fields	Avoidance of oil and gas fields		LU/LC map. 2024 (https://livingatlas.arcgis.com/landcover/)
Groundwater fields	Avoidance of groundwater fields	NA	
Water wells	Avoidance of water wells		Infrastructure map (https://www.google.com/maps/)

This study created a geospatial database that included data from digitized maps, remote sensing (RS) data, and statistical variables. Spatial modeling is based on functions and tools that combine spatial analysis and statistics. Spatial modeling was implemented after handling all the factors (applying Euclidean distance or equal-distance ranges and converting vector data to a grid raster) and obtaining a raster (pixels/cells) that expresses the current phenomenon. All raster maps used were reclassified before being added to the weighted overlay analysis. Reclassification helps determine the spatial ranges of typical work and defines them in categories 1–9 to standardize the scale (where all the criteria are reclassified to that scale, giving the most appropriate cells a value of 9 and the least appropriate value of 1). This process provides a new layer for each criterion added to the geographical databases. The balanced matching process requires that the criteria be standardized, enabling logical calculations to be performed on them when used as criteria within the model and ensuring the correctness and accuracy of the matching process.

GIS techniques provide a comprehensive view of their role in determining the most suitable sites and in linking all criteria affecting their identification. The GIS platform offers flexibility in assigning weights to these criteria, allowing a higher percentage to be allocated to a particular criterion based on its importance. This process is known as the fuzzy analytical hierarchy process (FAHP). FAHP is a combined multi-criteria technique developed by Mokarram and Hojati^56^, which evaluates the relevance of each factor at all locations and the importance of each component based on its unique position^23,24^. The FAHP methodology provides a diverse set of decision-making approaches spanning key dimensions. Consequently, the achieved resolution incorporates a degree of compromise between the selected criteria and the level of risk considered. This flexibility enables nuanced policymaking by considering different perspectives and risk scales^57^.

In the present study, the GIS-based MCDM-FAHP technique served as the primary framework for identifying suitable areas for wind farms in the region. This approach enables the calculation of layer-specific weights by accounting for each layer’s assigned importance levels. Consequently, a range of AHP operators, composed of well-known GIS-based map-combination techniques, can be created using different sets of order weights^25^. Examples of such operators are presented in Table 2 and Appendix 2.

#### Weighting methods of criteria

This section outlines the weight analysis criteria essential for making meaningful comparisons between studies. To ensure a robust and balanced weighting framework, the study employs an integrated multi-criteria weighting approach that combines the Fuzzy Analytical Hierarchy Process (FAHP), Entropy-based weighting, and Type-2 Fuzzy logic.

The relationships among these methods can be structured as follows: First, FAHP is used to capture expert preferences and address the inherent subjectivity of human judgment through linguistic variables. Simultaneously, the entropy-based weighting method is applied to the data to provide an objective measure of dispersion, ensuring that criteria with higher information diversity are assigned appropriate significance. Rather than using a single method to adjust the other in a linear sequence, the two sets of weights are combined to calculate a compromise weight (W_j).

Finally, Type-2 Fuzzy modeling is applied to the spatial aggregation process. While Type-1 fuzzy sets handle basic uncertainties, the Type-2 framework is specifically used to manage higher-level uncertainties arising from integrating diverse data sources (e.g., technical, environmental, and socioeconomic) and from potential variations in expert opinions. This integrated flow ensures that the final suitability index is neither purely subjective nor purely data-driven but a synthesis of both.

This compromise weight is then integrated into the GIS environment through Type-2 Fuzzy modeling during the weighted overlay process. Unlike standard overlay methods, Type-2 modeling handles ‘uncertainty about uncertainty’ by defining an interval-valued membership for each criterion (the footprint of uncertainty). The ArcGIS weighted overlay tool processes these intervals and applies the compromise weights to aggregate the layers. This multilayered approach ensures that the final wind suitability index for Kuwait accounts for both subjective and objective data variations while minimizing the impact of potential errors in the input datasets.

##### Fuzzy analytical hierarchy process (FAHP)

Pairwise comparisons and expert judgment were used to determine the relative importance of each component in relation to other factors. This technique prioritizes important criteria from the perspectives of multiple experts using FAHP and entropy-based aggregation. This approach can help legislators establish priorities and achieve desired results. It is a useful technique for overcoming challenging decision-making situations. FAHP replaces composite judgments with a series of pair-wise comparisons and findings, supporting both the subjective and objective components of a decision.

Furthermore, FAHP is a useful technique for assessing the accuracy of decision-makers’ evaluations and can help minimize decision-making bias. This is based on a pairwise comparison of the chosen criteria generated by experts’ evaluations. These opinions were used to ascertain the relative significance of the most important themes and their attributes. Because the comparisons were based on personal or subjective opinions, there may have been some disagreement. The ability to measure the consistency between paired comparisons across different criteria is one of the most significant advantages of the FAHP. This consistency ratio was found to confirm the consistency of perceptions. FAHP generates decisions as outputs after integrating geographic data as inputs. Qualitative data of numerous themes and attributes were transformed into quantitative data using Saaty’s scale to create a pairwise comparison matrix^58,59^. After consulting experts and specialists, appropriate weights for the natural and environmental conditions of the study area were adopted, and criteria that were not used in similar areas but were of a different kind in other areas were modified to fit the study area’s conditions.

##### Trapezoidal interval type II fuzzy analytical hierarchy process (T2-FAHP)

A Trapezoidal Interval Type-2 Fuzzy Analytical Hierarchy Process (TIT2-FAHP) method was presented by Gupta and Lee^60^. According to them, the trapezoidal interval type-2 fuzzy analytical hierarchy process (T2-FAHP) and FAHP are extensions of the classical AHP method designed to handle uncertainty and subjectivity in decision-making. However, they differ in their representations and in the uncertainties associated with their processes. Type-2 fuzzy set models vagueness and second-order uncertainty (uncertainty about the membership values themselves); is more robust, expressive, and suitable for highly uncertain or inconsistent expert inputs, and minimizes information loss owing to a richer representation of fuzziness. (As shown in Appendix 1)

The weights of the criteria used in the analysis were established as follows. The decision matrices contained expert responses based on the T2-FAHP (see Appendix 2); we converted these decision matrices into trapezoidal fuzzy integers^60,61^. Subsequently, the arithmetic mean of the respondents’ answers was determined using Eq. (1) to create an aggregated comparison matrix.

1 $$\:downright{Z}_{q}={\left({{\stackrel{\sim}{\stackrel{\sim}{x}}}_{ij}}^{q}\right)}_{m\times\:n}=\left[\begin{array}{ccc}{{\stackrel{\sim}{\stackrel{\sim}{x}}}_{11}}^{q}&\:\cdots\:&\:{{\stackrel{\sim}{\stackrel{\sim}{x}}}_{1n}}^{q}\\\: \vdots &\:\ddots\:&\: \vdots \\\:{{\stackrel{\sim}{\stackrel{\sim}{x}}}_{m1}}^{q}&\:\cdots\:&\:{{\stackrel{\sim}{\stackrel{\sim}{x}}}_{mn}}^{q}\end{array}\right],\:1\le\:i\le\:m,\:1\le\:j\le\:n,\:1\le\:q\le\:k$$

where $\:{\stackrel{\sim}{\stackrel{\sim}{x}}}_{ij}$ represents the upper and lower IT2 fuzzy sets’ aggregated matrix, which needs to be computed: $\:{\stackrel{\sim}{\stackrel{\sim}{\:x}}}_{ij}=\left(\frac{{{\stackrel{\sim}{\stackrel{\sim}{x}}}_{ij}}^{1}+{{\stackrel{\sim}{\stackrel{\sim}{x}}}_{ij}}^{2}+\dots\:+{{\stackrel{\sim}{\stackrel{\sim}{x}}}_{ij}}^{k}}{k}\right)$. Besides, $\:k\:$ refers to the number of respondents. Equations (2, 3) can be selected to obtain the upper ($\:{P}^{U}$) and lower ($\:{P}^{L}$) fuzzy matrices:

2 $$\:{P}^{U}=\left[\begin{array}{ccc}p({{\stackrel{\sim}{A}}_{1}}^{U}\ge\:{{\stackrel{\sim}{A}}_{1}}^{U})&\:\cdots\:&\:p({{\stackrel{\sim}{A}}_{1}}^{U}\ge\:{{\stackrel{\sim}{A}}_{n}}^{U})\\\: \vdots &\:\ddots\:&\: \vdots \\\:p({{\stackrel{\sim}{A}}_{n}}^{U}\ge\:{{\stackrel{\sim}{A}}_{1}}^{U})&\:\cdots\:&\:p({{\stackrel{\sim}{A}}_{n}}^{U}\ge\:{{\stackrel{\sim}{A}}_{n}}^{U})\end{array}\right],\:1\le\:i\le\:n$$

3 $$\:{P}^{L}=\left[\begin{array}{ccc}p({{\stackrel{\sim}{A}}_{1}}^{L}\ge\:{{\stackrel{\sim}{A}}_{1}}^{L})&\:\cdots\:&\:p({{\stackrel{\sim}{A}}_{1}}^{L}\ge\:{{\stackrel{\sim}{A}}_{n}}^{L})\\\: \vdots &\:\ddots\:&\: \vdots \\\:p({{\stackrel{\sim}{A}}_{n}}^{L}\ge\:{{\stackrel{\sim}{A}}_{1}}^{L})&\:\cdots\:&\:p({{\stackrel{\sim}{A}}_{n}}^{L}\ge\:{{\stackrel{\sim}{A}}_{n}}^{L})\end{array}\right],\:1\le\:i\le\:n$$

The upper membership ranking value function $\:Rank\left({{\stackrel{\sim}{A}}_{i}}^{U}\right)$, lower membership function $\:Rank\left({{\stackrel{\sim}{A}}_{i}}^{L}\right)$, and fuzzy set $\:Rank\:\left({\stackrel{\sim}{\stackrel{\sim}{A}}}_{i}\right)$ are calculated by use of Eqs. (4–6):

4 $$\:Rank\left({{\stackrel{\sim}{A}}_{i}}^{U}\right)=\frac{1}{n(n-1)}\left(\sum\limits_{k=1}^{n}p\left({{\stackrel{\sim}{A}}_{i}}^{U}\ge\:{{\stackrel{\sim}{A}}_{k}}^{U}\right)+\frac{n}{2}-1\right),\:1\le\:i\le\:n$$

5 $$\:Rank\left({{\stackrel{\sim}{A}}_{i}}^{L}\right)=\frac{1}{n(n-1)}\left(\sum\limits_{k=1}^{n}p\left({{\stackrel{\sim}{A}}_{i}}^{L}\ge\:{{\stackrel{\sim}{A}}_{k}}^{L}\right)+\frac{n}{2}-1\right),\:1\le\:i\le\:n$$

6 $$\:Rank\:\left({\stackrel{\sim}{\stackrel{\sim}{A}}}_{i}\right)=\frac{Rank\left({{\stackrel{\sim}{A}}_{i}}^{U}\right)+Rank\left({{\stackrel{\sim}{A}}_{i}}^{L}\right)}{2},\:1\le\:i\le\:n$$

where $$\:\sum\:_{i=1}^{n}Rank\:\left({\stackrel{\sim}{\stackrel{\sim}{A}}}_{i}\right)=1$$.

Lastly, the ranking values of the upper membership function are normalized using Eqs. (7, 8) ($\:{{d}_{ij}}^{U}$), lower membership function ($\:{{d}_{ij}}^{L}$). The average normalized ranking value of the fuzzy set is used to determine the relative weights ($\:{d}_{ij}$) is calculated using Eq. (9):

7 $$\:{{d}_{ij}}^{U}=\frac{Rank\left({{\stackrel{\sim}{A}}_{i}}^{U}\right)}{\sum\:_{j=1}^{J}Rank\left({{\stackrel{\sim}{A}}_{i}}^{U}\right)},\:1\le\:i\le\:n,\:1\le\:j\le\:J$$

8 $$\:{{d}_{ij}}^{L}=\frac{Rank\left({{\stackrel{\sim}{A}}_{i}}^{L}\right)}{\sum\:_{j=1}^{J}Rank\left({{\stackrel{\sim}{A}}_{i}}^{L}\right)},\:1\le\:i\le\:n,\:1\le\:j\le\:J$$

9 $$\:{d}_{ij}=\frac{{{d}_{ij}}^{U}+{{d}_{ij}}^{L}}{2}$$

where $\:{d}_{ij}$ indicates the preference rating of the criteria, and it shall be noted that $\:\sum\:{d}_{ij}=1$.

Type-2 fuzzy modeling was integrated into the GIS environment by treating each criterion as an interval-valued fuzzy set. The spatial overlay process calculated the intersection of the upper and lower membership boundaries for each pixel, which were then processed through a type-reduction algorithm to produce a robust crisp suitability index that minimizes the impact of data uncertainty.

#### MCDM suitability model according to the entropy-based aggregation approach

Gupta and Lee^60^ determined the weights for the criteria used in the analysis. Each criterion’s weighted average ($\:{{w}_{i}}^{avg}$) Eq. (10) can be used to define this.

10 $$\:{{w}_{i}}^{avg}=\frac{\sum\:_{j=1}^{m}{{w}_{i}}^{j}\times\:{S}_{j}}{\sum\:_{j=1}^{m}{S}_{j}}$$

where $\:{S}_{j}$ refers to the number of respondents in the j^th^ group.

Calculate the value of entropy ($\:{E}_{j}$) for every response group using Eq. (11):

11 $$\:{E}_{j}=\sum\limits_{i=1}^{n}\left(\frac{-1}{\mathrm{l}\mathrm{n}\left(2\right)}\left({{w}_{i}}^{j}\times\:\mathrm{ln}\left({{w}_{i}}^{j}\right)+{{w}_{i}}^{avg}\times\:\mathrm{l}\mathrm{n}\left({{w}_{i}}^{avg}\right)\right)\right)$$

where n is the number of criteria used.

The diversification value ($\:{div}_{j}$) for each correspondent and normalize these values ($\:{\stackrel{-}{w}}_{j}$) using Eqs. (12, 13) to get each group’s significance level:

12 $$\:{div}_{j}=n-{E}_{j}$$

13 $$\:{\stackrel{-}{w}}_{j}=\frac{{div}_{j}}{\sum\:_{j=1}^{m}{div}_{j}}$$

Compute the compromise weight ($\:{cw}_{i}$) of criteria and rank the estimated weights in descending order using Eq. (14) to identify the most important criteria:

14 $$\:{cw}_{i}=\sum\limits_{j=1}^{m}{\stackrel{-}{w}}_{j}\times\:{{w}_{i}}^{j}$$

In this process, it is essential to standardize suitability maps and assign weights based on their relative importance. The combination of weights and normalized suitability maps yielded the final suitability scores. The factor maps were categorized based on their weights, with first-class factors or those with higher preference degrees playing a crucial role in wind farm site selection. Subsequently, a multi-criteria evaluation and FAHP suitability maps for wind farms were developed. lists the designated criteria utilized as constraints, along with the corresponding threshold values and suitability mapping. This emphasizes the significance and feasibility of each independent criterion for mapping the suitability of wind farm sites. A scale ranging from 1 to 9 was used to assess fit, with 1 indicating the least favorable fit and 9 the most favorable. The conditioning factors were classified, and the data were stratified, as shown in Fig. 4. This process generates four layers: technical, topographic, economic, social, and environmental suitability. Subsequently, the optimal areas for the wind farms were determined by summing the four fundamental layers, as shown in Fig. 6.

### Criteria for location evaluation

Numerous considerations and restrictions are involved in determining the locations of wind farms in Kuwait. Consequently, the elements must be examined from multiple perspectives, including economic, technological, policy, and environmental. The ultimate criteria for this study were developed through a thorough literature analysis, consultation with similar research projects, and expert advice^28,29^. In any MCDM strategy, qualified experts must be included in any MCDM strategy. The most important aspect of this study is assigning relative weights to various criteria to determine their relative importance^31–33^. Therefore, a panel of five experienced professionals was engaged to identify the most critical criteria applicable to Kuwait, based on expertise and domain knowledge, using the FAHP to assess 26 important factors across four main groups: technical, topographic, economic, social, and environmental. The details of the various influential thematic maps generated, along with the criteria that affect wind farm suitability, are discussed in the following sections.

The five experts involved in the FAHP weighting process were strategically selected to represent the multidisciplinary nature of this study (Appendix 3). The panel comprised senior specialists from Kuwait University, KISR, and the EPA.

Regarding the number of experts, previous studies in MCDA have demonstrated that a small group of highly experienced experts (ranging from three to seven) is sufficient and often preferred to maintain consistency in pairwise comparisons, as shown in Appendix 3. For instance, Saaty^62^, the founder of AHP, noted that a large sample size is not required for this methodology. Furthermore, Darko et al.^63^ and Cheng and Li^64^ confirm that the quality and specialized knowledge of experts are more critical than quantity. In our study, the five selected experts represented the key stakeholder groups in Kuwait (academia, KISR, EPA, and industry), ensuring a comprehensive and authoritative perspective on the criterion weights, as presented in Appendix 2.

Expert judgments were collected via a structured pairwise-comparison questionnaire specifically designed for the FAHP framework. The process was conducted in three distinct stages:

1. *Briefing*: Each of the five experts received a technical brief outlining the study’s objectives, the definitions of each criterion (technical, topographic, socioeconomic, and environmental), and the 1-to-9 Saaty scale adapted for fuzzy linguistic variables.
2. *Data Collection*: Experts completed the matrices independently to avoid groupthink and ‘halo effects.’ The questionnaire required participants to compare the relative importance of criterion pairs (e.g., ‘Wind speed ‘vs. ‘Distance to grid ‘) using linguistic terms such as ‘Equally important,’ ‘Weakly important,’ or ‘Very strongly important.
3. *Consistency Check*: After collecting responses, the consistency ratio (CR) was calculated for each expert matrix to assess the mathematical reliability of the resulting weights. Any matrix exceeding the 0.1 threshold was returned to the expert for a structured review and adjustment to ensure logical consistency, after which the responses were aggregated using the geometric mean to obtain the final FAHP weights. The *CR* values for the pairwise comparison matrices in this study are as follows: (*Expert 1*:0.081, *Expert 2*:0.074, *Expert 3*:0.069, *Expert 4*:0.088, and *Expert 5*:0.072), indicating that the pairwise comparisons provided by the expert panel are logically consistent and suitable for further multi-criteria decision-making analysis.

#### Technical criteria

Technical factors play a significant role in determining potential wind energy sites, accounting for approximately 40% of the total criterion weights (Appendices 2 and 4). Within this factor, the average wind speed (measured in m/s) directly affects wind farm performance, particularly turbine efficiency. Numerous studies have highlighted that average wind speeds below 6 m/s are not economically viable for wind energy projects. Consequently, the average wind speed was reclassified based on the study area characteristics. In our analysis, we considered wind speeds exceeding 7 m/s at a height of 50 m to be the optimal minimum threshold for wind generation (This value is not only a technical benchmark for modern utility-scale turbines but also aligns with the specific atmospheric conditions and previous research conducted in the Arabian Peninsula). The selection of a wind speed threshold of > 7 m/s at a height of 50–100 m is grounded in both turbine performance characteristics and regional meteorological assessments. From a technical standpoint, most modern Class I and Class II wind turbines—designed for the medium-to-high wind regimes typical of Kuwait’s open desert—reach their ‘rated power’ (maximum efficiency) at wind speeds between 7 m/s and 12 m/s. While many turbines have a ‘cut-in’ speed as low as 3–4 m/s, power generation at those levels is insufficient for commercial viability^53,65^.

To facilitate this, we categorized wind speed into 9 groups, all of which exceeded 7 m/s (Fig. 2a). Another crucial parameter for wind energy projects is wind power density, which is calculated from average wind speed and air density in the region.

The wind power density was derived as follows^66^:

7 $$\:\rho\:=1.225-\left(1.194\cdot\:{10}^{-4}\:h\right)$$

8 $$\:WPD=\frac{1}{2}\rho\:{V}^{3}$$

where WPD is wind power density (W/m^2^), V is the average wind speed (m/s); $\:\rho\:$ is the air density (kg/m^3^), and $\:h$ is elevation (m).

**Fig. 5:**
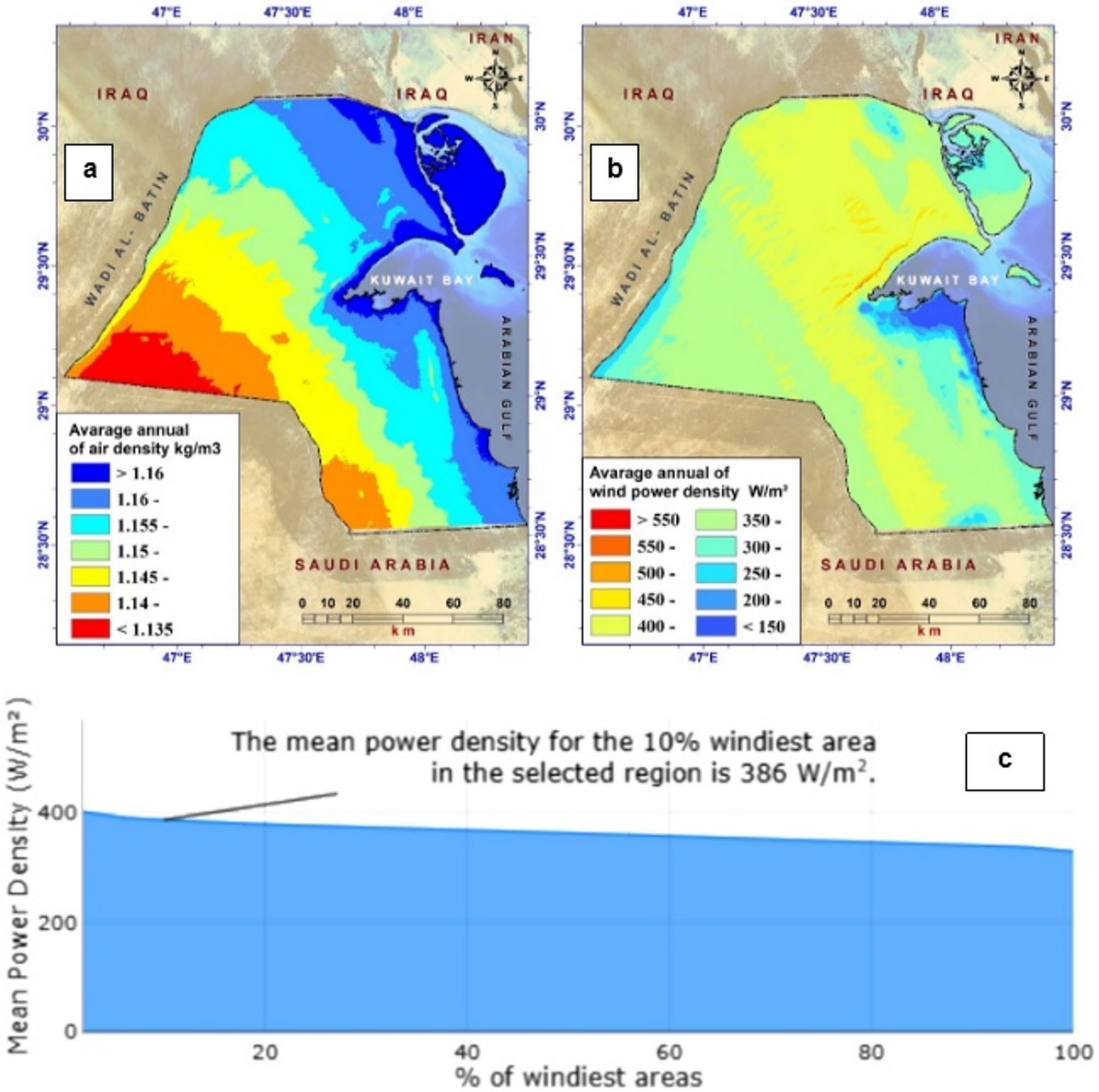
**a** Annual air density (kg/m^3^) over Kuwait at 50 m height; **b** annual wind power density (W/m^2^) over Kuwait at 50 m height; and **c** mean power density (W/m^2^) diagram^46^.

Figure 5 shows spatial variations in air and wind power density (WPD) across the country. WPD decreased as one approached Kuwait Bay, whereas it increased in expansive flat desert areas, such as Al-Wafra, Al-Taweel, and Umm Omara. The calculations revealed that the annual WPD ranged from 590.46 to 70.73 W/m². This can be attributed to a decrease in air density along the shores of the Gulf of Kuwait and an increase in air density in the western desert regions. The annual air density was determined to be between 1.130 and 1.162 kg/m³. When selecting wind farm sites, proximity to transmission lines and main power stations is a crucial consideration. Several studies suggest that a distance of at least 250–500 m from high-voltage lines is suitable for the proposed locations^53,66^. To account for this factor, we considered nine distance categories ranging from 500 to 5000 m (Appendices 4D and 4E).

#### Topographic criteria

This study focused on evaluating the topographical characteristics of wind power stations, which accounted for approximately 30% of the total weight of the criteria (see Appendices 2 and 5). These characteristics encompass land inclinations that affect wind turbine performance. Numerous studies have demonstrated that steep slopes are unfavorable for wind power generation because the wind does not strike the turbine rotor at an optimal angle, thereby increasing fatigue. Typically, slopes exceeding 5° can lead to turbulent wind patterns that compromise turbine stability (see Fig. 3a). Moreover, constructing steeper slopes increases project costs. Ideally, the terrain should be flat or gently rounded, as it would be exposed to higher and more consistent wind speeds^39^.

Additionally, the direction of slope inclinations can influence the identification of suitable sites for establishing wind power stations. Following the approach in Section^67^, this study categorized wind speed classes by shaded and sunny directions: north, northeast, northwest, and east; and south, southeast, west, and northwest (see Fig. 3b). However, aspect and slope had minimal effects on the localization of optimal wind farms in the current case study. This study introduced its maps as a preliminary step towards the optimal exploitation of other renewable energies and strategic planning within the study area. As depicted in Fig. 3c and d, our study avoided proposing wind power generation sites in areas characterized by loose limestone soils and Quaternary geological deposits from the Pleistocene era. Such areas typically exhibit numerous valleys, sabkhas, and salt marsh deposits, which can pose long-term risks and potential damage to wind power stations.

#### Social and economic (socio-economic) criteria

Socioeconomic criteria represented 20% of the total weight of the standards (see Appendix 6). Among these criteria is proximity to roads. The distance between the proposed farm site and the road network should be minimized to reduce the cost of constructing wind farm sites. We considered a distance larger than 500 m between roads and the location of the stations inappropriate, according to previous studies^68^, where the raster layer, which indicates the distance between the optimal sites and the main roads, has been reclassified into nine categories, with values ranging between 500 and 5000 m (see Appendix 6(g)). The distance from urban, industrial, and military areas is an important criterion for selecting wind farm locations; therefore, buffer distances must be established to avoid noise, disturbance, and impacts on the natural environment.

Therefore, wind farms should be located within 2000 m of the city to enable efficient energy transmission, reduce infrastructure costs, and meet urban energy demand. This minimizes the environmental impact and increases public acceptance. Moreover, areas more than 50 km from the inhabited centers were considered inappropriate, as presented in Appendices 6(a), (b), and (c). The distance to airports is also important when determining suitable wind farm sites. This is because tall wind turbines can affect navigation, and electromagnetic interference from wind farms can affect radar and flight paths to airports near the proposed sites. Moreover, it is impossible to fly in the area closest to the wind turbines, as a buffer distance of more than 3 km and less than 3 km from the airports, according to previous studies^53^ (see Appendix 6(f)).

#### Environmental criteria

Environmental factors are important considerations when constructing wind power stations for electricity generation. This category encompasses factors such as distance from beaches, protected areas, river drainage networks, and movement rates of high dunes. Consequently, in this study, these environmental factors accounted for 10% of the overall weight of the criteria (see Appendices 2 and 7). Based on Moiloa’s^69^ recommendations, it is advisable to maintain a distance of approximately 4 km between wind farms and the coast to account for bird flight paths and future tourism activities. Accordingly, we established a buffer distance of 500 m to separate wind farms from beaches and wetlands^53^(see Appendix 7 (a)).

When considering protected areas, it is crucial to maintain a distance between wind farm sites and these areas, which may include inhabited regions, historical sites, bird habitats, and migration routes. The point layer representing protected areas was classified into nine categories with heights greater than 10 km. In addition, other significant geological and geomorphological sites and nature reserves are protected under national legislation. These standards serve as limitations because the development of wind farms can significantly affect the environmental value of these areas (see Appendix 7(b)). This study also considered the ecological threats associated with the construction of wind energy stations. We defined nine categories with equal standard distances, with the first indicating an unsuitable site and the ninth the most suitable. These threats encompass sand movement, drainage network density, seismic hazards, seismicity distribution, and fault density (see Appendices 7(c), (d), (e), (f), and (g)). Land cover and use play a crucial role in the development of wind energy. Certain land types are unfavorable for wind power stations, including saline soils, water bodies, agricultural areas, dunes, urban areas, and regions with groundwater wells, oil, and natural gas, as shown in Appendices 6 and 7.

In summary, the methodology followed a structured four-stage workflow. First, spatial data were gathered and preprocessed within a GIS environment. Second, entropy–based weighting and FAHP were applied to determine the relative importance of each criterion. Third, Type-2 fuzzy modeling was used to aggregate these criteria while accounting for data uncertainty. Finally, a suitability map was generated and validated against Kuwait’s strategic energy targets, as detailed in the flowchart (Fig. 4).

## Results

**Fig. 6:**
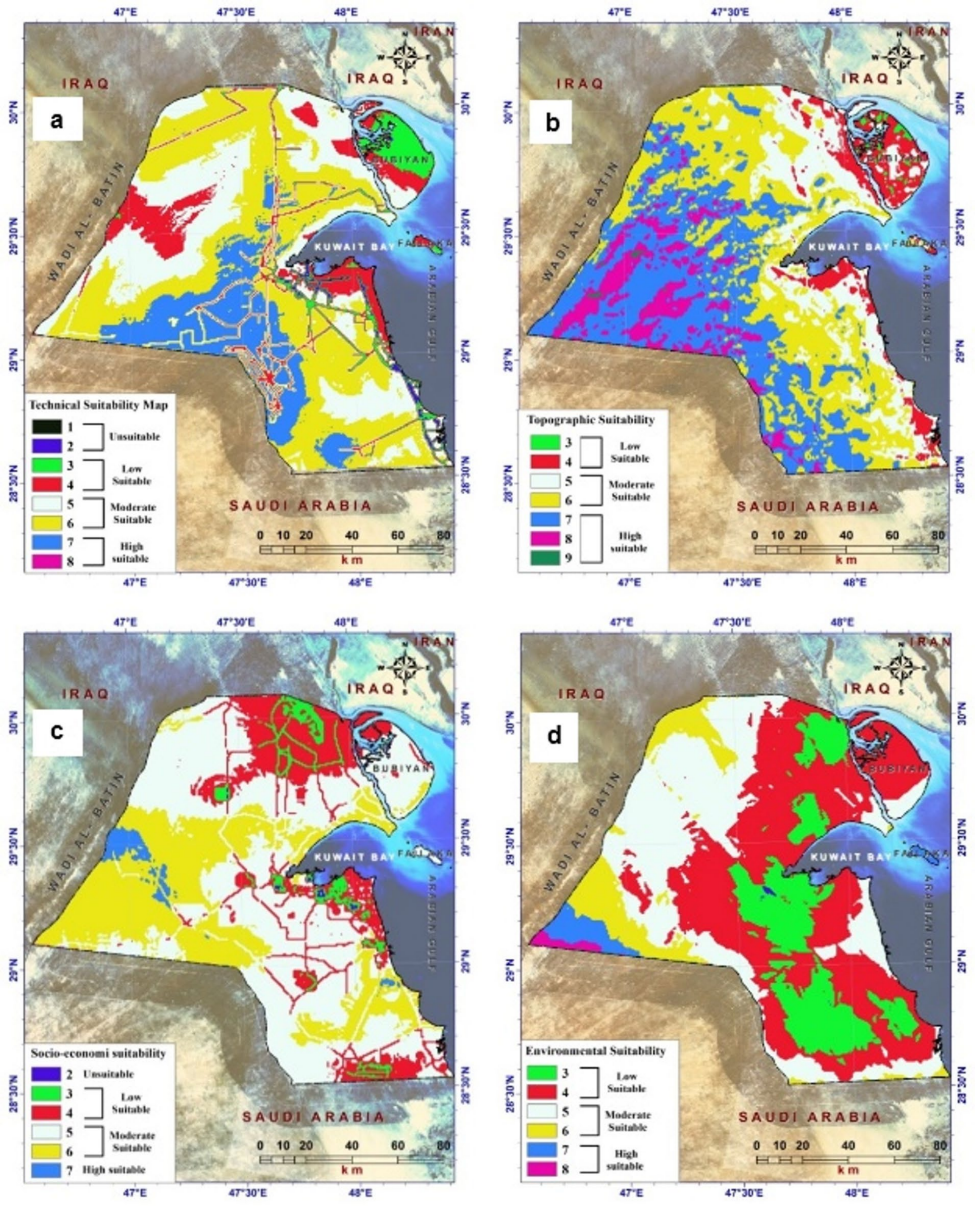
Suitability map criteria for wind farms: **a** technical suitability map, **b** topographic suitability map, **c** social and economic suitability map, and **d** environmental suitability map.

**Fig. 7:**
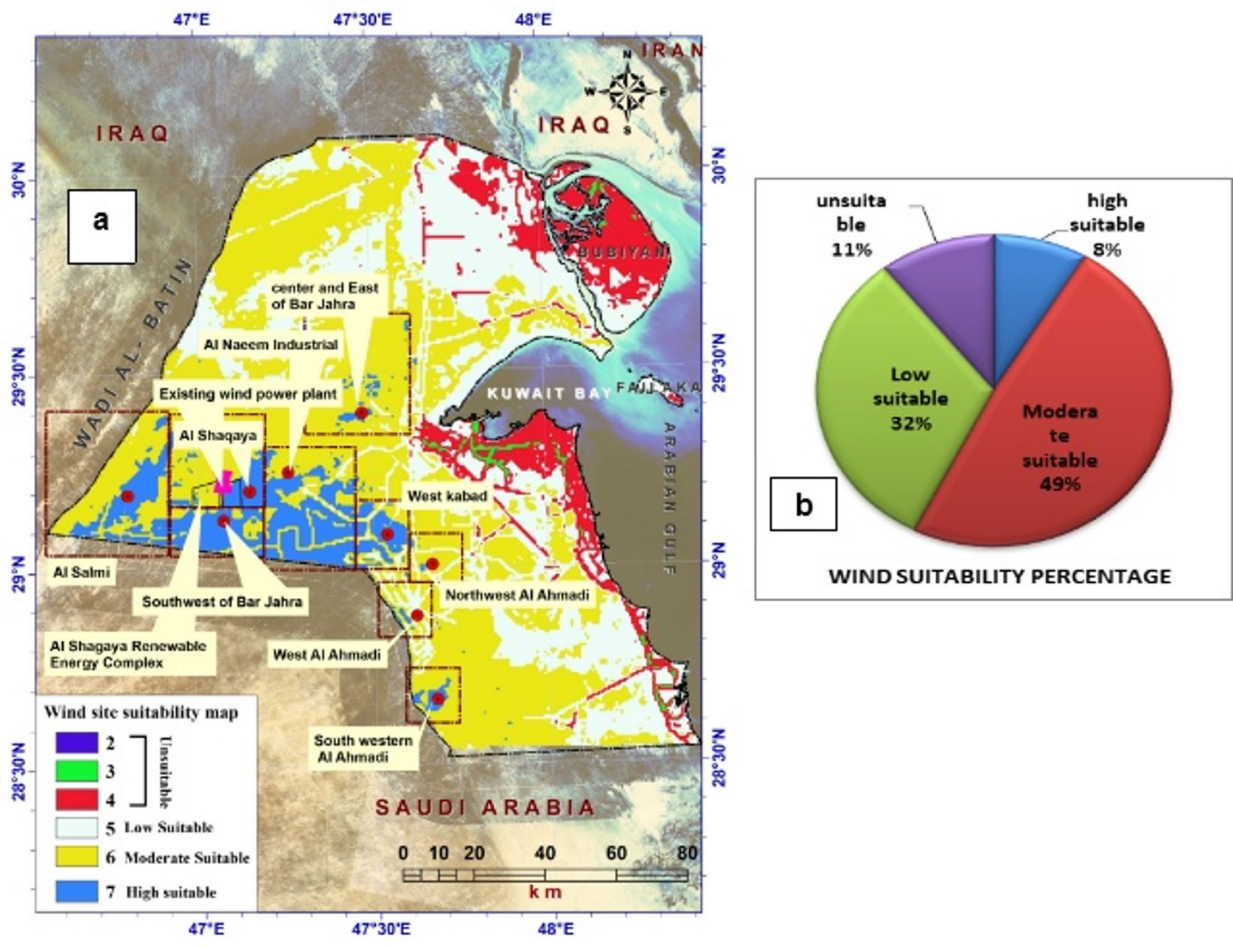
**a** Highly Potential Zones for Wind Farm Planting in Kuwait and **b** their Expected Percentages.

Based on these criteria, our methodology identified the most favorable sites for wind farm construction. We employed weighted overlay analysis in ArcGIS to merge all datasets. The resulting output was a map highlighting suitable areas, determined from the weighted overlay analysis of the technical, topographic, social, economic, and environmental criteria (Fig. 6 and Appendix 2). The final map depicting wind energy suitability encompassed four distinct categories: unsuitable, low suitability, moderate suitability, and high suitability (Fig. 7; Table 3).

### Evaluation of suitability criteria for site selection of wind farms

The suitability map for wind farms, generated using the GIS-based MCDM technique, is presented in Fig. 7 and depicted in Fig. 4. The calculated weights were derived using the FAHP method, as listed in Table 2. The suitability analysis yielded four distinct layers: technical, topographic, economic, social, and environmental. The technical suitability map (Fig. 6a) shows the spatial suitability resulting from the weighted overlay of the five criteria that contribute to and indicate wind energy suitability in Kuwait. These criteria include wind speed, transmission lines, power stations, wind power density, and air density (see Appendix 1). Among these factors, the highest weights were assigned to wind speed and power density, as they play a crucial role in turbine movement and capture a substantial amount of wind energy for efficient electricity generation. The technical suitability map was categorized into 8 classes, reflecting the integration and coherence of the layers within the technical suitability model used to identify wind farm areas in Kuwait. Values 1 and 2 represent problematic areas, while values 7 and 8 represent highly suitable areas. These characteristics are particularly evident in regions such as West Jahra, Al Ahmadi, and the nearby Al-Wafra farms. This can be attributed to higher wind speeds, greater wind energy intensity, reduced air density, and proximity to power lines and power stations in these areas.

The topographic suitability map shows the spatial suitability of wind energy in Kuwait (Fig. 6b). This map was generated by conducting a weighted overlay analysis of five factors that contribute to wind energy: elevation (m), slope (%), aspect, geology, and soil (Fig. 3). Among these factors, elevation (m), aspect, and slope (%) received the highest weights because of their significant roles in preparing the terrain for wind power plants and enhancing wind generation capacity. The topographic suitability map was divided into seven categories by integrating and aligning the layers used in the topographic suitability model to identify wind farm locations in Kuwait. Values 3, 4, and 7 represent areas with poor suitability, while values 7, 8, and 9 indicate high suitability. These high-suitability areas are prominent in West Jahra, Al Ahmadi, and the nearby Al-Wafra farms. This can be attributed to factors such as increased elevation, terrain level, alignment of slopes with the dominant winds in the region, and the natural suitability of soil formation in those areas.

The socioeconomic suitability map assesses the spatial suitability of wind energy in Kuwait, as shown in Fig. 6c. This map was generated by conducting a weighted overlay analysis of nine criteria that contribute to wind energy suitability: proximity to urban areas (cities), roads, airports, military areas, oil and gas fields, groundwater fields, agricultural areas, industrial areas, salt lakes, and swamps (Appendix 5). Among these criteria, proximity to airports received the highest weight owing to its significant and direct impact on the preparation of power plant sites, turbine installation, and operational range. The socioeconomic suitability map was divided into six categories based on the integration and alignment of the layers within the socioeconomic suitability model used to identify wind farm areas in Kuwait. Values 2 and 7 indicate inappropriate and extremely suitable locations, respectively. These highly suitable locations and prepared sites for optimal wind energy efficiency are predominantly found in West Jahra. These areas were chosen to be free of residential and commercial activities, as well as lakes and swamps, ensuring an open, unobstructed environment for wind energy generation.

The environmental suitability map shows the spatial suitability of wind energy in Kuwait (Fig. 6d). This map was generated by conducting a weighted overlay analysis of nine criteria contributing to wind energy suitability: proximity to sand movement, drainage network density, fault density, seismicity distribution, seismic hazard, protected areas, and shorelines (Appendix 7). Among these criteria, proximity to protected areas and shorelines received the highest weight because of their significant and direct impact on the preparation of power plant sites and turbine installation. The environmental suitability map was divided into 8 categories based on the integration and alignment of layers in the environmental suitability model used to determine wind farm areas in Kuwait. A value of 3 indicated low suitability, and 8 indicated high suitability. Notably, these highly suitable areas, particularly in West Jahra, were chosen for their distance from environmental and geological risks that could hinder the establishment of renewable-energy facilities. This ensures that wind energy stations are located in areas free from such threats, thereby promoting the successful implementation of wind energy projects.

Figure 7 visually represents the zones with high potential for wind farm development in Kuwait along with their expected percentages. This figure complements the information presented in Table 3, which outlines the distribution of land suitable for wind farms in the region: 8.6% of the total land area (1,444.48 km²) is classified as highly suitable, which likely corresponds to the highly potential zones shown in Fig. 7. These zones represent the best areas for optimal wind energy generation. Additionally, the table shows that 48.79% (8,192.29 km²) of the land is moderately suitable for wind farm development, whereas 31.99% (5,370.78 km²) is unsuitable. Only 10.62% (1,783.05 km²) of the land was classified as unsuitable for wind farm establishment. Together, the figure and table highlight that a substantial portion of Kuwait’s land is either highly or moderately suitable for wind energy projects, indicating promising opportunities for sustainable energy development in these regions.

**Table 3:** Percentage of land suitability areas for wind farms.

Suitability classes	Zones description	Area (Km^2^)	%
High suitable	Optimal	1444.48	8.6
Moderate suitable	Viable	8192.29	48.79
Low suitable	Marginal	5370.78	31.99
Unsuitable	Restricted	1783.05	10.62
Total		16790.6	100

### Technical estimation of generation capacity

The estimated generation capacity of 2,500 MW is based on a conservative net land utilization of 35% in the ‘Highly Suitable’ zones. By applying a standard power density of 5 MW/km^2,^ the model confirms that these regions can comfortably host the infrastructure required to meet nearly 60% of Kuwait’s 2030 renewable energy target (approximately 4500 MW total). 4500 MW total). The calculation followed a conservative estimation model commonly used in regional wind energy assessments:

1. *Effective Land Area*: The total area identified as ‘Highly Suitable’ is approximately 1,444.48 km². To account for internal spacing between turbines (to minimize wake effects) and logistical constraints, a ‘net utilization factor’ of 5% to 10% of this total area was considered for actual turbine placement.
2. *Power Density Assumption*: Based on the performance specifications of modern utility-scale wind turbines (such as those used in the Shagaya project) and the average wind power density in western Kuwait, a conservative power density of 5 MW per km² was applied.
3. *Calculation*: By applying these parameters to the core high-potential zones–specifically, the contiguous tracts in the Jahra and Al-Abali regions the model indicates that, even with a low-density array configuration, the area can comfortably support an installed capacity of 2,500 MW.

This estimation serves as a baseline for national energy planners, suggesting that the identified regions can significantly contribute to Kuwait’s goal of generating 15% of their peak demand from renewable sources by 2030.

## Discussion

**Fig. 8:**
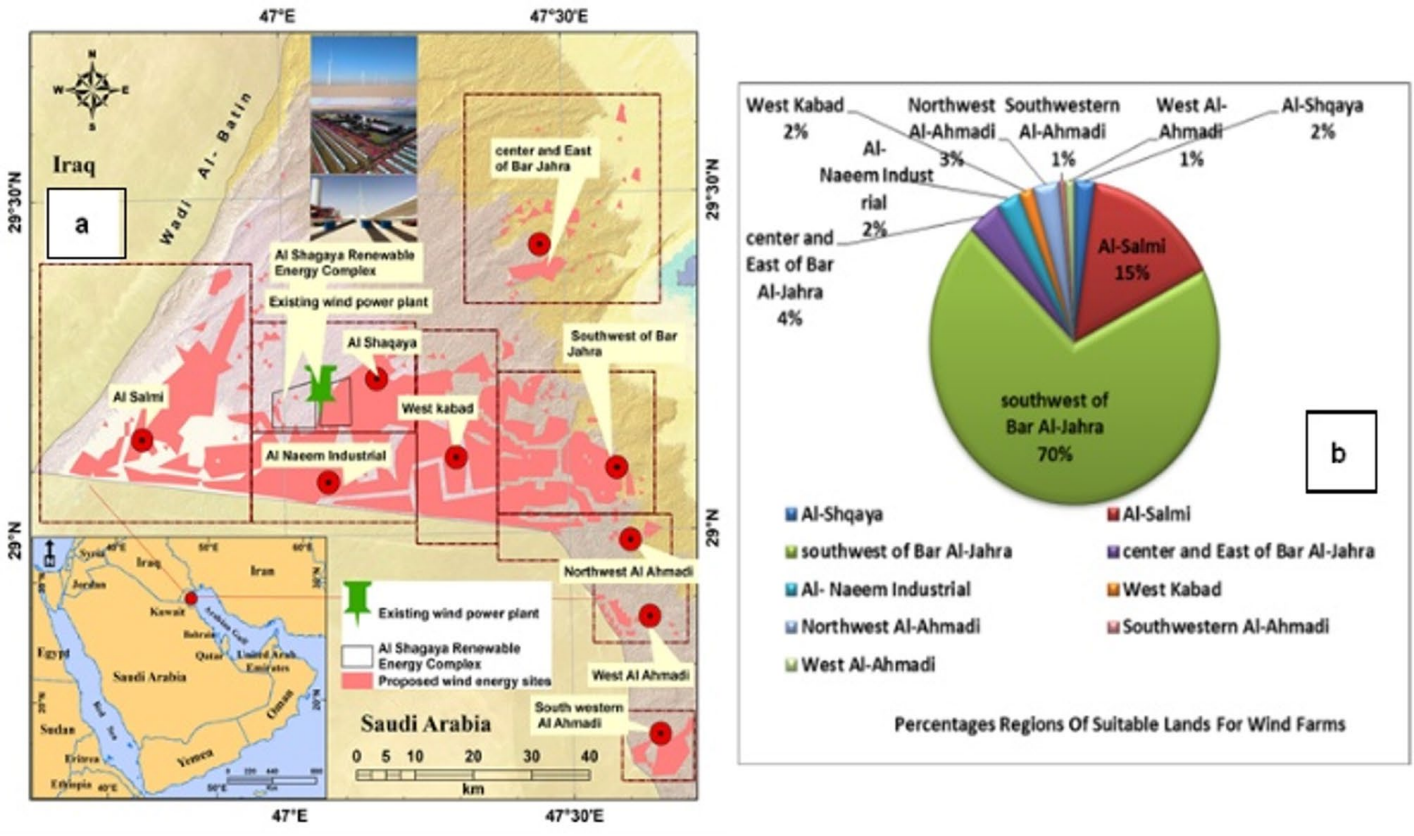
**a** Wind suitability map in Kuwait using GIS-based MCDM-AHP of the integrated factors and **b** their expected percentages.

The outcomes derived from the weighted multi-criteria overlay analysis, as illustrated in Fig. 8, provide insights into the wind suitability map for Kuwait. The map highlights the expected percentages and identifies the most suitable areas in the western regions, such as the Al-Jahra and Al-Ahmadi governorates. These areas offer favorable conditions for establishing wind farms, with proximity to transmission lines and high wind speeds. However, as we move towards the eastern part of the study area, wind energy suitability gradually diminishes due to lower wind speeds. Eight optimal regions were identified within these preferred zones as prime locations for establishing wind farms. These regions include Al-Shqaya, Al-Salmi, the southwest of Bar Al-Jahra, the center and east of Bar Al-Jahra, Al-Naeem Industrial, West Kabad, Northwest Al-Ahmadi, and West Al-Ahmadi. These locations offer abundant wind resources and are advantageous because of their proximity to major highways, power lines, and urban areas.

The analysis demonstrates that the most favorable locations for establishing wind farms are primarily situated west of the Al-Jahra Governorate, as outlined in Table 4. Nearly 95.32% of this governorate’s total area is suitable for wind farm installation. Another promising site lies in the southwest region of Bar Al-Jahra, encompassing approximately 69.76% of the area, with altitudes ranging from 190 to 230 m and an average wind speed of 6.66 m/s. Conversely, the least suitable area was West Kabad, which constituted only 1.61% of the land area. This region ranges in altitude from 137 to 154 m and has an average wind speed of 7 m/s. approximately 4.67% of the total area suitable for wind energy generation was attributed to the Al-Ahmadi Governorate. Additionally, the study highlights that the central and eastern parts of Bar Al-Jahra house have the highest energy-producing regions in Kuwait, with a production capacity of 411 W/m2. In contrast, the Al-Salmi area exhibited the lowest energy production of 309 W/m2.

**Fig. 9:**
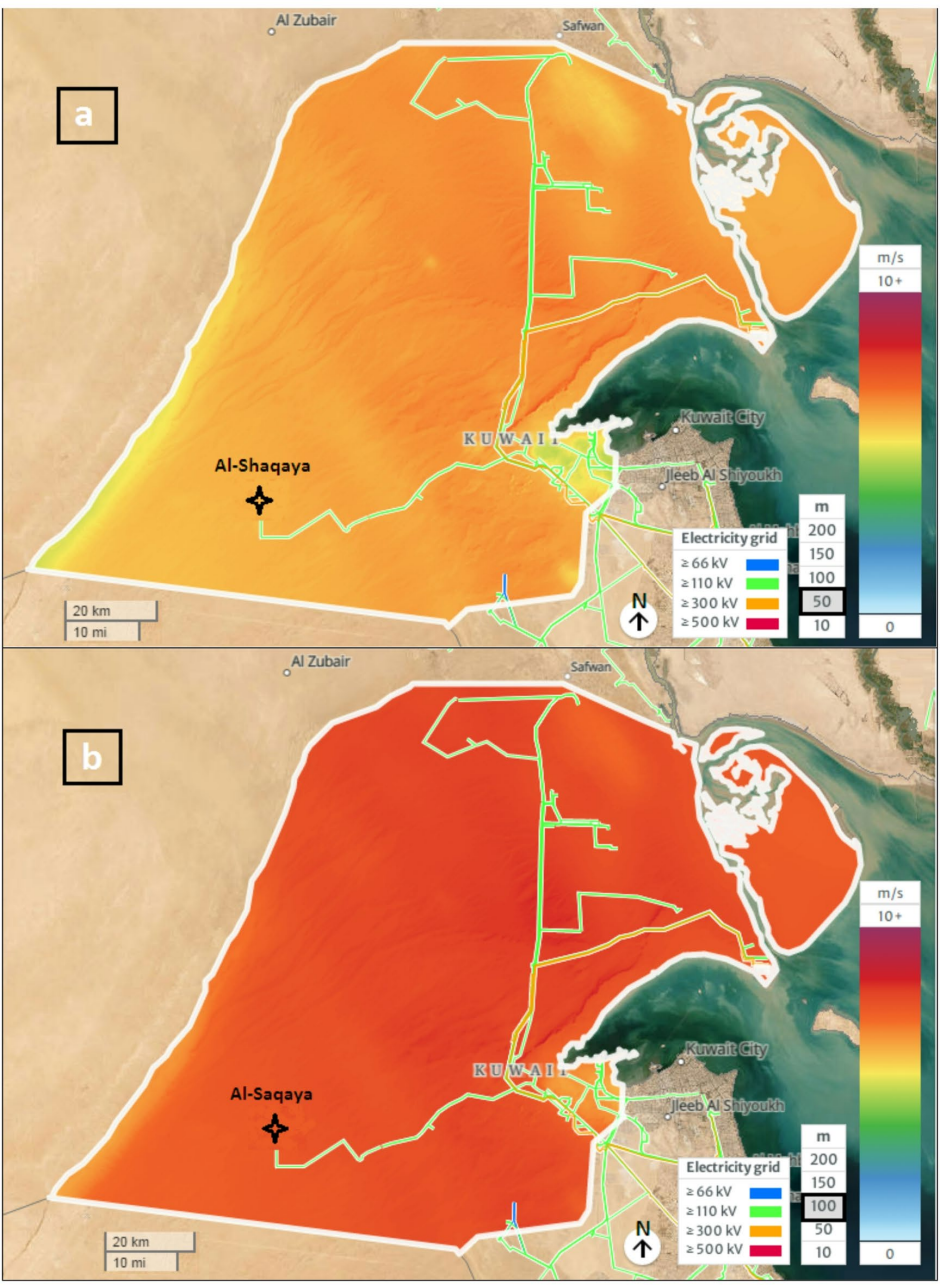
Wind speed and electricity grid from the (GWA 3.0) dataset: **a** At 50 m (a. s. l.) and **b** At 100 m (a. s. l.)

Figure 8 shows the zones with the highest potential for establishing wind power plants, as identified by this study’s analysis. To validate our findings, we included the Shagaya wind power plant in western Kuwait. It is recognized as the first renewable energy plant in the Arabian Gulf region to combine wind, photovoltaic, and concentrated solar power. The location of Shagaya aligns with one of the high-potential zones identified in this study (infrastructure overlay, Fig. 9). The results confirmed that 100% of the existing turbine locations fell within the ‘Highly Suitable’ (Class 4) zone, which is characterized by optimal wind speeds and minimal topographic constraints. This alignment demonstrates the model’s ability to identify commercially viable sites that have already passed rigorous government feasibility studies. This validation adds credibility to our results and demonstrates the practical applicability of our proposed methodology. Based on these findings, we strongly recommend conducting further studies to evaluate other high-potential zones. This would enable a comprehensive understanding of Kuwait’s broader opportunities to expand its renewable energy initiatives. In addition, the final suitability map was cross-referenced with the Global Wind Atlas (GWA 3.0) dataset (Fig. 9a and b) to calculate the statistical correlation. A pixel-by-pixel correlation analysis was conducted between the model’s suitability index and the mean wind power density values from the Global Wind Atlas at a 50- and 100-m hub height. The spatial distribution of ‘Highly Suitable’ areas in the western (Al-Abali) and northern (Al-Jahra) regions showed a strong correlation (R² = 0.84) with the highest wind-power-density corridors identified by the GWA. This dual validation confirms that the integrated FAHP–entropy approach effectively filters out unsuitable terrain while accurately capturing the most productive wind regimes in Kuwait’s desert environment.

It is important to note that the locations selected in this study are preliminary, and further comprehensive research is required before a final decision can be made. The GIS-based approach employed in this study facilitated the quantitative evaluation of variables and constraints to be considered when identifying optimal wind farm locations. The generated suitability map is a valuable tool for guiding the search for potential new wind farm sites in the region. However, it is crucial to emphasize that on-site measurements and field investigations should be conducted to gather additional data before making the final decision on constructing a wind farm. Furthermore, it is advisable to verify the final map’s findings by measuring wind speeds and observing other relevant ecological variables in each region to assess their suitability for wind farm placement. This step ensures a more accurate evaluation of potential sites and improves decision-making.

According to the spatial analysis, western Kuwait (specifically the Al-Jahra and Al-Abali regions) constitutes the most significant ‘highly suitable’ corridor for wind energy development. This concentration of potential can be attributed to several key factors:

**Table 4:** Percentage of regions and energy production (w/m^2^) of suitable land for wind farms.

Governorate	Region	Area km^2^	%	Height (m)	Annual average wind speed (m/s)	Air density	Power density (w/m^2^)
Al-Jahra	Al-Shqaya	33.32	2.31	215–260 m	6.52	1.133	322
	Al-Salmi	220.67	15.3	228–255 m	6.43	1.132	309
	southwest of Bar Al-Jahra	1007.41	69.8	190–230 m	6.66	1.14	336
	center and East of Bar Al-Jahra	55.05	3.81	158	7.21	1.143	411
	Al-Naeem Industrial	36.98	2.56	187–225 m	7	1.138	340
	West Kabad	23.32	1.62	137–154 m	7	1.141	366
	Total	1376.76	95.3				2084
Al-Ahmadi	Northwest Al-Ahmadi	42.82	2.96	152	6.83	1.144	341
	Southwestern Al-Ahmadi	9.40	0.65	184	6.89	1.136	359
	West Al-Ahmadi	15.20	1.05	168	6.73	1.141	325
	Total	67.42	4.67				1025

1. *Optimized Wind Regimes*: According to the GWA and local KISR meteorological data, western Kuwait experiences a ‘channeling effect’ of the prevailing North-Westerly (Shamal) winds. As these winds move across the flat, open desert of the Arabian interior toward the coast, they maintain higher speeds and lower turbulence because of the lack of topographic obstructions. The mean wind speed at 100 m in these zones consistently exceeds 7.5 m/s, providing a high energy density compared to the more shielded eastern or southern coastal areas.
2. *Topographic uniformity*: The western interior is characterized by a stable, flat-to-sloping plateau with minimal elevation changes. This reduces the complexity of turbine foundation engineering and minimizes wake losses between turbine rows. The low slope (generally < 3%) in these areas is ideal for the heavy machinery required during construction and maintenance.
3. *Minimal Land-Use Conflict*: Unlike the eastern and coastal regions, which are densely populated and host critical national infrastructure, the western desert is largely undeveloped. Applying the 26-criteria model determined that the west offers the largest contiguous tracts of land outside of urban buffers (5,000 m), oil field-restricted zones, and highly sensitive coastal ecosystems.
4. *Proximity to Strategic Infrastructure*: Although remote, these western zones are traversed by high-voltage transmission lines connecting the Shagaya power complex to the national grid. This proximity significantly reduces the levelized cost of energy (LCoE) by minimizing the need for new, long-distance transmission infrastructure.

The high-suitability zones identified in our model, particularly in the northern and western corridors, are consistent with the early findings of Al-Nassar et al. ^19^. Their analysis of ground-based stations confirmed that Kuwait’s wind regime is characterized by a strong seasonal correlation, with peak power density occurring during the summer months when electricity demand is at its maximum. By confirming these historical trends through modern geospatial modeling and multi-criteria decision-making (MCDM), this study validates the long-term reliability of these regions for utility-scale energy production.

“Interestingly, the high suitability trends observed in the northern coastal fringes of our onshore model align with the offshore potential identified by Hasan et al. ^39^. This spatial synergy suggests that the northern corridor of Kuwait (from Al-Abali to the coast) represents a ‘Mega-Wind Zone’ where both onshore and future offshore projects could be integrated into a unified energy hub, maximizing the efficiency of the national grid connection.”

### Wind energy potential in kuwait: analysis and strategic considerations

The assessment of wind energy potential in Kuwait using GIS and remote sensing techniques has significant implications for policymakers and energy planners, particularly considering the country’s growing energy demands. The findings suggest that approximately 8% of Kuwait’s land area is suitable for wind farms, particularly in the Al-Jahra and Al-Ahmadi governorates. The strategic implementation of wind energy projects at identified sites can help diversify Kuwait’s energy portfolio, reduce its reliance on fossil fuels, and align with global efforts to address climate change^8,70^.

Local policy recommendations include developing detailed resource maps to guide planning, establishing regulatory frameworks to attract private investment, and enhancing the electrical grid to accommodate renewable energy sources. Additionally, raising public awareness of the benefits of wind energy and fostering community involvement in renewable energy projects are crucial. Future research should focus on the economic feasibility of wind projects, long-term performance monitoring, interdisciplinary approaches to integrate various renewable energy sources, and the assessment of the impacts of climate change on wind energy generation in Kuwait. However, limitations such as data availability, static modeling assumptions, and the need to consider socioeconomic factors may affect the outcomes of such studies^71,72^.

The findings indicate that the areas in Kuwait most suitable for wind energy are primarily concentrated in the western and northern desert plateaus. When compared to similar studies in the Gulf Cooperation Council (GCC) region, such as those conducted in Saudi Arabia (e.g., Al-Yahyai et al.)^53^ and Oman (e.g., Albadi et al.)^54^ (Table 5), a distinct geographical trend emerges. While Saudi Arabian suitability is often dictated by high-altitude, mountainous terrain in the Sarawat range, Kuwait’s suitability is driven by the flat, unobstructed fetch of the Arabian Desert, which allows for stable, laminar wind flow despite lower overall elevations.

Furthermore, the application of fuzzy AHP in this study provides a more nuanced transition between the ‘suitable’ and ‘unsuitable’ zones compared to the ‘crisp’ Boolean logic used in previous regional assessments. For instance, in Jordan’s arid regions, traditional AHP studies often overlook the fuzzy nature of environmental constraints, such as sand dune migration. By integrating fuzzy membership, this study acknowledges that the impact of Kuwait’s dust corridor is a gradient rather than a fixed boundary. This aligns with the findings of the Shagaya Renewable Energy Park phase I^55^, confirming that the exclusion of oil concession areas and military zones remains a more significant limiting factor for Kuwait than for its neighbors, where topography and social acceptance are the primary constraints.

**Table 5:** Results comparison (Examples from GCC). Source: After^73–76^.

Feature/Criteria	Current study (Kuwait)	Saudi Arabia	Jordan	Oman
Topography	Flat Desert Plateaus	High Altitude/Escarpments	Mountainous/Desert Mix	Coastal Cliffs & Plains
Wind Speed Range	6.2–7.8 m/s	7.5–9.0 m/s	6.5–8.5 m/s	7.0–8.2 m/s
Methodology	Fuzzy AHP + GIS	Standard AHP + GIS	WLC/Boolean Logic	Multi-Criteria (MCDA)
Primary Constraint	Oil Concessions & Dust	Topography & Grid Access	Urban encroachment	Marine Protection Zones
Critical Risk Factor	Sand Dune Migration	Turbulence/Wake Effect	Social acceptance	Humidity / Salt Corrosion

### Alignment and policy implications with Kuwait’s renewable energy strategies

The results of this suitability model directly support Kuwait’s national strategic objectives and energy policy, particularly as the state strives to transition toward a more diversified and sustainable energy portfolio, including the mandate to generate 15% of the country’s electricity from renewable sources by 2030 as a green energy target. The model aligns with the following national pillars:

1. *Strategic Land Allocation and Zoning*: The identification of 1,444 km² of ‘Highly Suitable’ land provides a spatial blueprint for the Kuwait Municipality and the MEW. Policymakers can use these results to establish ‘renewable energy zones’ (REZs), ensuring that land is legally protected from urban encroachment or industrial competition. This proactive zoning is essential to streamline the licensing process for future international investors.
2. *Supporting Kuwait Vision 2035 (New Kuwait)*: The identification of optimal sites in the Jahra and Ahmadi governorates provides a spatial roadmap for the ‘Sustainable Living Environment’ pillar of the national vision. By focusing development in these high-potential zones, the state can maximize the return on investment (ROI) for infrastructure. In addition, the model’s results offer technical validation for the ‘New Kuwait’ 2035 mandate. By confirming that 8.6% of the country’s land is highly viable for wind energy, the study proves that the national goal of 15% renewable energy is not only ambitious but also geographically and technically attainable. This evidence can be used to secure higher budget allocations for Phases II and III of the Shagaya Renewable Energy Park.
3. *Shqaya Renewable Energy Park Master Plan*: Our model independently confirms that the Shqaya region is among the most viable corridors in the country. These findings support the planned expansion of Phases II and III of the Shagaya project, suggesting that the western desert corridor remains the most technically sound location for large-scale wind integration.
4. *Land Use Optimization*: The model incorporates 26 distinct criteria, including proximity to urban centers and protected areas, to ensure that renewable energy expansion does not conflict with future urban residential growth or sensitive desert ecosystems. This ‘conflict-free’ siting approach is essential for the Kuwait Municipality and the EPA in their long-term land allocation processes.
5. *Infrastructure and Grid Integration*: Because the most suitable zones are concentrated in the west and north, there is a clear policy need to prioritize expanding high-voltage transmission networks toward these regions. Policymakers should focus on ‘grid-readiness’ to ensure that when large-scale wind farms are constructed, the energy can be efficiently evacuated to the main load centers in Kuwait City and Jahra with minimal transmission losses.
6. *Economic Diversification and Carbon Commitments*: Developing the identified wind-rich corridors will help Kuwait reduce its domestic reliance on liquid fuels for power generation. This has a dual economic benefit: it increases the volume of oil available for export and helps Kuwait meet its Nationally Determined Contributions (NDCs) under the Paris Agreement. Transition to wind energy in these high-potential zones is a critical step in reducing the national carbon footprint.
7. *Environmental and Regulatory Harmony*: This study provides a framework for ' pre-vetting” sites by integrating 26 environmental and socioeconomic constraints. This reduces the time and cost of *environmental impact assessments (EIAs)* for developers, as the model has already accounted for protected areas, migratory bird paths, and sensitive desert ecosystems, ensuring a smoother regulatory approval process.

## Conclusions

Kuwait’s energy demand has increased owing to rapid population growth. To address this, Kuwait has implemented a program over the past decade to establish solar photovoltaic (PV) and wind power plants as alternative sources of renewable energy to meet its energy requirements. This strategic plan has effectively bridged the gap between energy supply and demand, while simultaneously reducing thermal emissions. This study aimed to assess Kuwait’s wind energy potential and examine its prospects by integrating remotely sensed spatial modeling data and GIS-based multi-criteria decision analysis–AHP techniques as a comprehensive assessment approach, integrating multiple criteria to identify the most suitable areas for wind energy installations. The findings of this study highlight the substantial wind power generation potential across extensive regions in western Kuwait, particularly in the Al-Jahra and Al-Ahmadi governorates.

The findings indicate that approximately 8% of Kuwait’s total land area is suitable for wind farm development. These areas are primarily concentrated in Al-Jahra and Al-Ahmadi, accounting for 95.32% and 4.67% of the total area, respectively. Within these regions, eight specific locations are highly recommended for harnessing wind energy: Al-Shqaya, Al-Salmi, southwest of Bar Al-Jahra, center, and east of Bar Al-Jahra, Al-Naeem Industrial, West Kabad, Northwest Al-Ahmadi, West Al-Ahmadi, and Southwestern Al-Ahmadi. These locations possess advantageous technical, topographic, economic, social, and environmental characteristics that are conducive to wind energy development. Furthermore, this study identifies significant research gaps that should be addressed to implement large-scale renewable electricity projects successfully. A crucial aspect is the need for detailed resource maps that encompass various renewable energy generation technologies and that focus on their technical and economic potential. Addressing these gaps would enable the revision of master plans for energy complexes, thereby enabling a more accurate calculation of the substantial contribution of renewable energy to the country’s overall energy framework. The overall strategy provides a systematic method that guides planners through the necessary steps for combining multiple criteria, incorporating expert opinions on the criteria and their respective weights.

Future research should explore the feasibility of offshore wind potential along Kuwait’s coastline to capitalize on higher wind speeds. Additionally, investigating hybrid renewable systems that integrate wind and solar energy could provide a more stable power supply for the national grid. Finally, assessing the long-term impacts of climate variability on wind patterns is essential to ensure the resilience and sustainability of wind energy investments in the region.

## Supplementary Information

Below is the link to the electronic supplementary material.

Supplementary Material 1.

Supplementary Material 2.

Supplementary Material 3.

## Data Availability

The datasets used and/or analyzed in the current study are available from the corresponding author upon reasonable request.

## Abbreviations

MCDM: Multi-criteria decision making
FAHP: Fuzzy analytic hierarchy process
SDG 7: Sustainable Development goal 7 (affordable and clean energy)
GIS: Geographic Information Systems
TOPSIS: Technique for order of preference similarity to ideal solution
Fuzzy TOPSIS: Fuzzy extension of TOPSIS
DEA: Data envelopment analysis
WASPAS: Weighted aggregated sum-product assessment
SWARA: Stepwise weight assessment ratio analysis
MCE: Multi criteria evaluation
KISR: Kuwait Institute for Scientific Research
MEW: Kuwait Ministry of Electricity and Water
NPC: Kuwait National Petroleum Company
IDW: Inverse distance weighting
LULC: Land use and land cover
OSM: OpenStreetMap
SRTM-DEM: Shuttle radar topography mission-digital elevation model
MCA: Multi-criteria analysis
EPA: Kuwait Environment Public Authority
WMCA: Weighted multi-criteria analysis
RS: Remote sensing
TIT2-FAHP: Trapezoidal interval type-2 fuzzy analytical hierarchy process
CR: Consistency ratio
WPD: Wind power density
GCC: Gulf Cooperation Council
GWA: Global Wind Atlas
