# Supplementary material for: Integrating fuzzy AHP and geo-spatial modeling for wind farm suitability assessment in Kuwait
Source: Sci Rep. 2026 Apr 3;16:11601. doi: 10.1038/s41598-026-46695-4 (PMC13056916; doi:10.1038/s41598-026-46695-4)
Supplement: Supplementary file 1 — Supplementary Material 1. [file 41598_2026_46695_MOESM1_ESM.docx]

# Analytic Hierarchy Process (AHP) Analysis

This table presents a full Analytic Hierarchy Process (AHP) analysis for evaluating site suitability based on technical, topographic, socio-economic, and environmental factors. Each category contains sub-factors with assigned importance levels and weight factors. The overall evaluation integrates these weights to assess site viability.

## Technical

| **Factor** | **Importance** | **Weight Factor %** | **Normalized Weight** | **Importance Criteria** | **Weight**  **%** |
| --- | --- | --- | --- | --- | --- |
| Average wind speed | 1 | 23 | 0.0920 | 1 | 40 |
| Transmission lines | 2 | 20 | 0.0800 |  |  |
| Power station | 3 | 17 | 0.0680 |  |  |
| Power density | 1 | 23 | 0.0920 |  |  |
| Air density | 3 | 17 | 0.0680 |  |  |

## Topographic

| **Factor** | **Importance** | **Weight Factor %** | **Normalized Weight** | **Importance Criteria** | **Weight %** |
| --- | --- | --- | --- | --- | --- |
| Elevation (m) | 1 | 31 | 0.0930 | 2 | 30 |
| Slope (Degree) | 2 | 25 | 0.0750 |  |  |
| Aspect | 3 | 20 | 0.0600 |  |  |
| Geological formation | 4 | 12 | 0.0360 |  |  |
| Soil types | 4 | 12 | 0.0360 |  |  |

## Socio-economic

| **Factor** | **Importance** | **Weight Factor %** | **Normalized Weight** | **Importance Criteria** | **Weight %** |
| --- | --- | --- | --- | --- | --- |
| Proximity to an urban area (cities) | 2 | 11 | 0.0220 | 3 | 20 |
| Proximity to roads | 2 | 11 | 0.0220 |  |  |
| Proximity to airports | 1 | 14 | 0.0280 |  |  |
| Proximity to military areas | 2 | 11 | 0.0220 |  |  |
| Proximity to oil and gas fields | 3 | 10 | 0.0200 |  |  |
| Proximity to groundwater fields | 3 | 10 | 0.0200 |  |  |
| Proximity to agricultural areas | 2 | 11 | 0.0220 |  |  |
| Proximity to industrial areas | 2 | 11 | 0.0220 |  |  |
| Proximity to salt lakes and swamps | 2 | 11 | 0.0220 |  |  |

## Environmental

| **Factor** | **Importance** | **Weight Factor %** | **Normalized Weight** | **Importance Criteria** | **Weight %** |
| --- | --- | --- | --- | --- | --- |
| Sand movement | 3 | 12 | 0.0120 | 4 | 10 |
| Proximity to drainage network density | 3 | 12 | 0.0120 |  |  |
| Proximity to fault density | 2 | 14 | 0.0140 |  |  |
| Seismic hazard | 2 | 14 | 0.0140 |  |  |
| Proximity to seismicity distribution | 2 | 14 | 0.0140 |  |  |
| Proximity to protected areas | 1 | 17 | 0.0170 |  |  |
| Proximity to shoreline | 1 | 17 | 0.0170 |  |  |
