# Supplementary material for: Integrating fuzzy AHP and geo-spatial modeling for wind farm suitability assessment in Kuwait
Source: Sci Rep. 2026 Apr 3;16:11601. doi: 10.1038/s41598-026-46695-4 (PMC13056916; doi:10.1038/s41598-026-46695-4)
Supplement: Supplementary file 2 — Supplementary Material 2. [file 41598_2026_46695_MOESM2_ESM.docx]

**Type-2 Trapezoidal Fuzzy Numbers**

**Step 1: Map Importance Values to Interval Type-2 Trapezoidal Fuzzy Numbers**

We'll define the membership functions for importance values (1–4). Each fuzzy number has:

- An **upper membership function (UMF)** and a **lower membership function (LMF)**.
- Format: UMF: (a1, a2, a3, a4), LMF: (b1, b2, b3, b4)


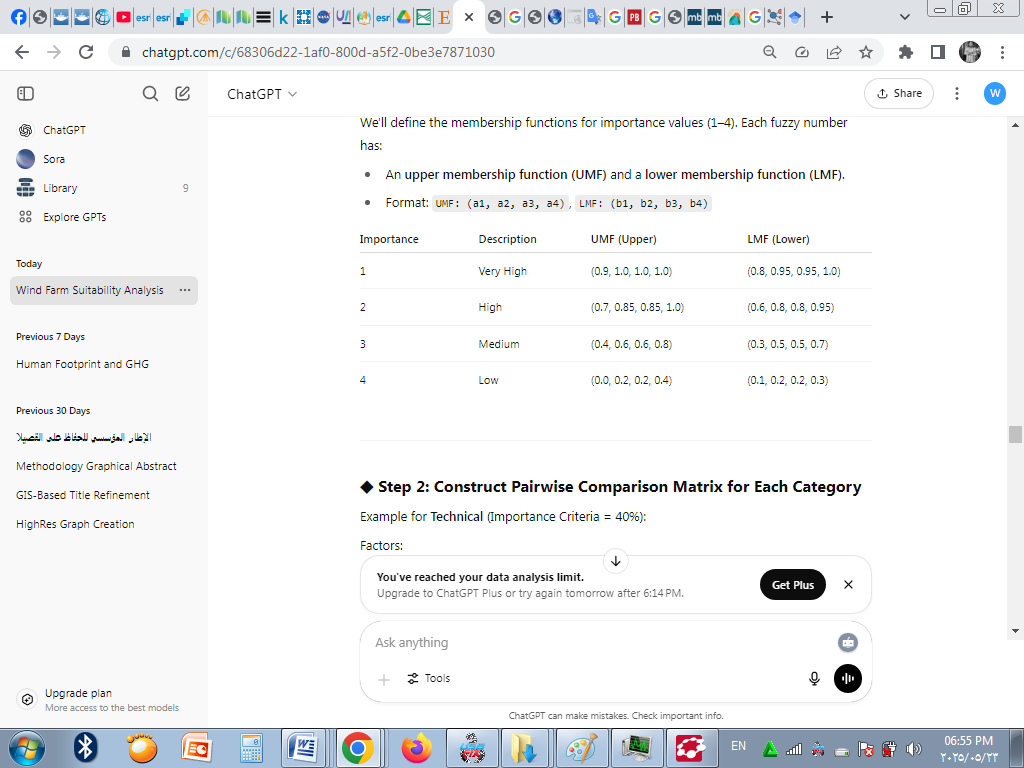


### ****Step 2: Construct Pair wise Comparison Matrix for Each Category****

Example for **Technical** (Importance Criteria = 40%):

Factors:

1. Average wind speed (1)
2. Transmission lines (2)
3. Power station (3)
4. Power density (1)
5. Air density (3)

Each pair wise comparison entry A_ij is calculated as: Aij=Fuzzy importance of i÷Fuzzy importance of jA_{ij} = \text{Fuzzy importance of } i \div \text{Fuzzy importance of } jAij​=Fuzzy importance of i÷Fuzzy importance of j

To divide fuzzy numbers: inverse the trapezoid and apply fuzzy multiplication.

This yields a **5×5 fuzzy matrix** with entries like:

AWS TL PS PD AD

AWS 1 >1 >>1 1 >>1

TL <1 1 >1 <1 >1

...

Where “>1” implies relative importance > 1 based on fuzzy arithmetic.

### ****Step 3: Fuzzy Synthesis / Weight Aggregation****

Sum the fuzzy numbers row-wise and normalize:

- Si=∑jAijS_i = \sum_j A_{ij}Si​=∑j​Aij​
- Then normalize: Wi=Si/∑iSiW_i = S_i / \sum_i S_iWi​=Si​/∑i​Si​ (fuzzy division)

### ****Step 4: Type Reduction (e.g., Karnik-Mendel Algorithm)****

Convert each fuzzy weight (an IT2FN) to a crisp number by:

1. Finding centroid of the lower and upper membership functions.
2. Average both to get final crisp weight.

Example:

- Lower: (0.6, 0.7, 0.7, 0.8), centroid ≈ 0.7
- Upper: (0.7, 0.8, 0.8, 0.9), centroid ≈ 0.8
- Final weight ≈ (0.7 + 0.8) / 2 = **0.75**

### ****Step 5: Global Weights Calculation****

Multiply each sub-factor's local weight by the **Importance Weight %** of its category:

For example, if “Power Density” gets 0.25 from Step 4, and the Technical category is weighted 0.4 (40%):

- Final weight = 0.25×0.4=0.100.25 \times 0.4 = 0.100.25×0.4=0.10
