# Supplementary material for: Integrating fuzzy AHP and geo-spatial modeling for wind farm suitability assessment in Kuwait
Source: Sci Rep. 2026 Apr 3;16:11601. doi: 10.1038/s41598-026-46695-4 (PMC13056916; doi:10.1038/s41598-026-46695-4)
Supplement: Supplementary file 3 — Supplementary Material 3. [file 41598_2026_46695_MOESM3_ESM.docx]

**Appendix**

**Appendix 1 : Type2-FAHP method**

**Type-2 Trapezoidal Fuzzy Numbers**

**Step 1: Map Importance Values to Interval Type-2 Trapezoidal Fuzzy Numbers**

We'll define the membership functions for importance values (1–4). Each fuzzy number has:

- An **upper membership function (UMF)** and a **lower membership function (LMF)**.
- Format: UMF: (a1, a2, a3, a4), LMF: (b1, b2, b3, b4)


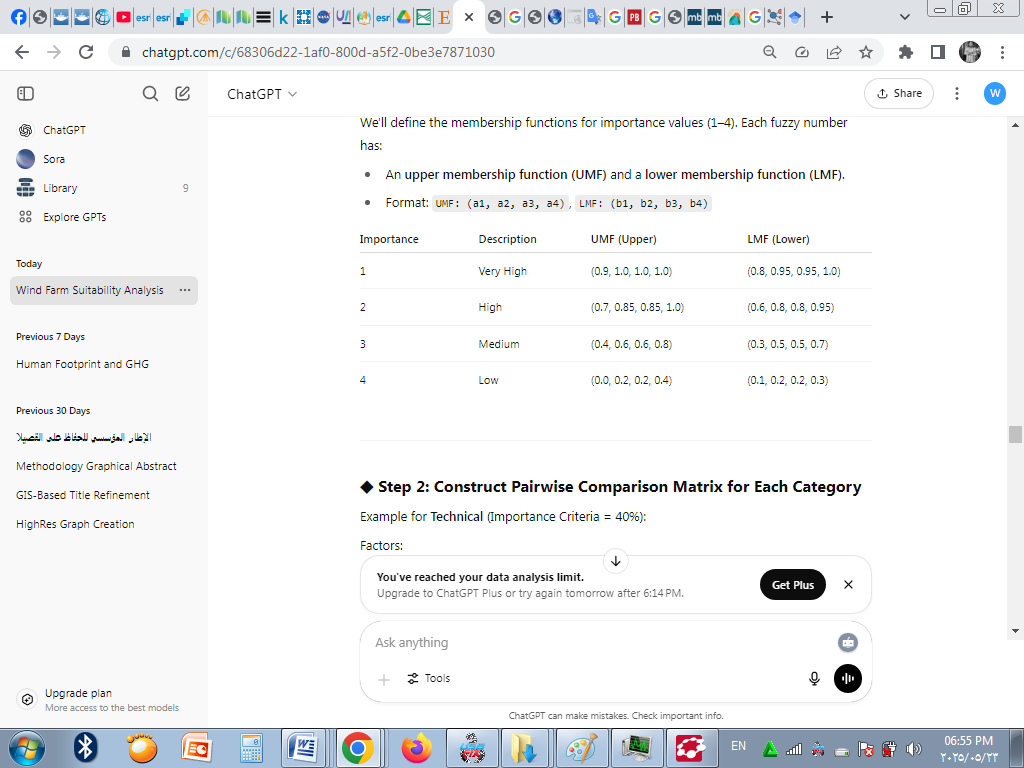


### ****Step 2: Construct Pairwise Comparison Matrix for Each Category****

Example for **Technical** (Importance Criteria = 40%):

Factors:

1. Average wind speed (1)
2. Transmission lines (2)
3. Power station (3)
4. Power density (1)
5. Air density (3)

Each pair wise comparison entry A_ij is calculated as: Aij=Fuzzy importance of i÷Fuzzy importance of jA_{ij} = \text{Fuzzy importance of }i \div \text{Fuzzy importance of }jAij​=Fuzzy importance of i÷Fuzzy importance of j

To divide fuzzy numbers: inverse the trapezoid and apply fuzzy multiplication.

This yields a **5×5 fuzzy matrix** with entries like:

AWS TL PS PD AD

AWS 1 >1 >>1 1 >>1

TL <1 1 >1 <1 >1

...

Where “>1” implies relative importance > 1 based on fuzzy arithmetic.

### ****Step 3: Fuzzy Synthesis / Weight Aggregation****

Sum the fuzzy numbers row-wise and normalize:

- Si=∑jAijS_i = \sum_j A_{ij}Si​=∑j​Aij​
- Then normalize: Wi=Si/∑iSiW_i = S_i / \sum_iS_iWi​=Si​/∑i​Si​ (fuzzy division)

### ****Step 4: Type Reduction (e.g., Karnik-Mendel Algorithm)****

Convert each fuzzy weight (an IT2FN) to a crisp number by:

1. Finding the centroid of the lower and upper membership functions.
2. Average both to get the final crisp weight.

Example:

- Lower: (0.6, 0.7, 0.7, 0.8), centroid ≈ 0.7
- Upper: (0.7, 0.8, 0.8, 0.9), centroid ≈ 0.8
- Final weight ≈ (0.7 + 0.8) / 2 = **0.75**

### ****Step 5: Global Weights Calculation****

Multiply each sub-factor's local weight by the **Importance Weight %** of its category:

For example, if “Power Density” gets 0.25 from Step 4, and the Technical category is weighted 0.4 (40%):

- Final weight = 0.25×0.4=0.100.25 \times 0.4 = 0.100.25×0.4=0.10

### Appendix 2: Weighting criteria used in the wind farm suitability analysis

### 2.1 Technical

| **Factor** | **Importance** | **Weight Factor %** | **Normalized Weight** | **Importance Criteria** | **Weight**  **%** |
| --- | --- | --- | --- | --- | --- |
| Average wind speed | 1 | 23 | 0.0920 | **1** | **40** |
| Transmission lines | 2 | 20 | 0.0800 |  |  |
| Power station | 3 | 17 | 0.0680 |  |  |
| Power density | 1 | 23 | 0.0920 |  |  |
| Air density | 3 | 17 | 0.0680 |  |  |

**2.2 Topographic**

| **Factor** | **Importance** | **Weight Factor %** | **Normalized Weight** | **Importance Criteria** | **Weight**  **%** |
| --- | --- | --- | --- | --- | --- |
| Elevation (m) | 1 | 31 | 0.0930 | **2** | **30** |
| Slope (Degree) | 2 | 25 | 0.0750 |  |  |
| Aspect | 3 | 20 | 0.0600 |  |  |
| Geological formation | 4 | 12 | 0.0360 |  |  |
| Soil types | 4 | 12 | 0.0360 |  |  |

**2.3 Socio-economic**

| **Factor** | **Importance** | **Weight Factor %** | **Normalized Weight** | **Importance Criteria** | **Weight**  **%** |
| --- | --- | --- | --- | --- | --- |
| Proximity to an urban area (cities) | 2 | 11 | 0.0220 | **3** | **20** |
| Proximity to roads | 2 | 11 | 0.0220 |  |  |
| Proximity to airports | 1 | 14 | 0.0280 |  |  |
| Proximity to military areas | 2 | 11 | 0.0220 |  |  |
| Proximity to oil and gas fields | 3 | 10 | 0.0200 |  |  |
| Proximity to groundwater fields | 3 | 10 | 0.0200 |  |  |
| Proximity to agricultural areas | 2 | 11 | 0.0220 |  |  |
| Proximity to industrial areas | 2 | 11 | 0.0220 |  |  |
| Proximity to salt lakes and swamps | 2 | 11 | 0.0220 |  |  |

**2.4 Environmental**

| **Factor** | **Importance** | **Weight Factor %** | **Normalized Weight** | **Importance Criteria** | **Weight**  **%** |
| --- | --- | --- | --- | --- | --- |
| Sand movement | 3 | 12 | 0.0120 | **4** | **10** |
| Proximity to drainage network density | 3 | 12 | 0.0120 |  |  |
| Proximity to fault density | 2 | 14 | 0.0140 |  |  |
| Seismic hazard | 2 | 14 | 0.0140 |  |  |
| Proximity to seismicity distribution | 2 | 14 | 0.0140 |  |  |
| Proximity to protected areas | 1 | 17 | 0.0170 |  |  |
| Proximity to shoreline | 1 | 17 | 0.0170 |  |  |

### Appendix 3: Profile of Experts Involved in the Fuzzy Multi-Criteria Decision Analysis

The determination of criteria weights was supported by a panel of five experts selected to provide a balanced perspective on wind energy development in Kuwait. The selection was based on three primary criteria: (i) a minimum of 10 years of professional or academic experience in their respective fields, (ii) a proven track record in renewable energy projects or geospatial analysis within the Gulf Cooperation Council (GCC) region, and (iii) a relevant postgraduate degree or senior technical role in government or industry.

The panel's diverse backgrounds ensured that technical, environmental, and socio-economic factors were weighted with a holistic understanding of the local context. The group included a Senior GIS Specialist with expertise in spatial modeling, a Renewable Energy Researcher focused on wind turbine efficiency, an Environmental Impact Assessment (EIA) Consultant specializing in desert ecosystems, an Urban Planner familiar with Kuwait’s land-use regulations, and a Civil Engineer with experience in large-scale infrastructure projects. This multidisciplinary composition allowed the FAHP and entropy-based weighting to account for both theoretical ideals and practical implementation challenges in Kuwait’s unique environment.

Experts profile table:

| Expert ID | Area of Expertise | Years of Experience | Affiliation | Rationale for Selection |
| --- | --- | --- | --- | --- |
| Expert 1 | GIS & Spatial Decision Support | 18 | Academic (Kuwait University) | - Multidisciplinary Insight: They bridge the gap between theoretical research (Academic) and practical implementation (Government/Industry) across GIS, renewable energy, and environmental policy. - Local Context Mastery: With an average of over 15 years of experience, these experts possess deep knowledge of Kuwait’s unique environmental constraints and regulatory framework. - Objectivity: The inclusion of different affiliation types ensures a balanced weighting of criteria, minimizing individual bias and enhancing the credibility of the fuzzy pair wise comparison results. |
| Expert 2 | Renewable Energy Engineering | 20 | Government Research (KISR) |  |
| Expert 3 | Environmental Impact Assessment | >12 | Regulatory Authority (EPA) |  |
| Expert 4 | Urban and Infrastructure Planning | 15 | Public Sector (Municipality) |  |
| Expert 5 | Energy Economics and Policy | >10 | Industrial Consultant |  |


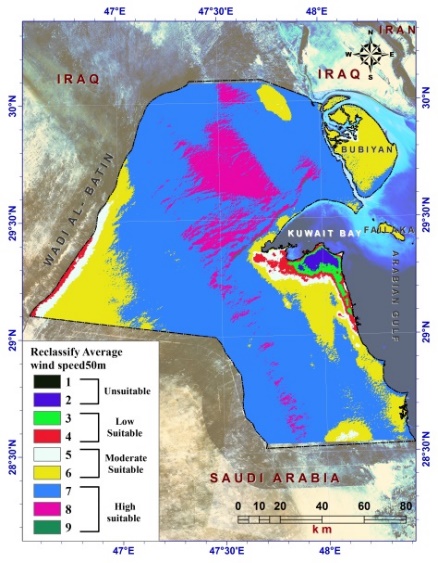

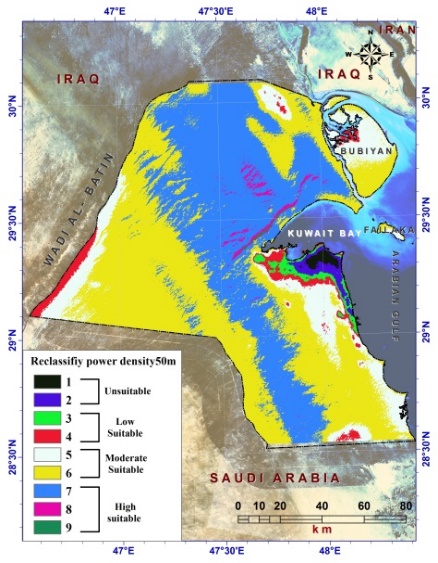

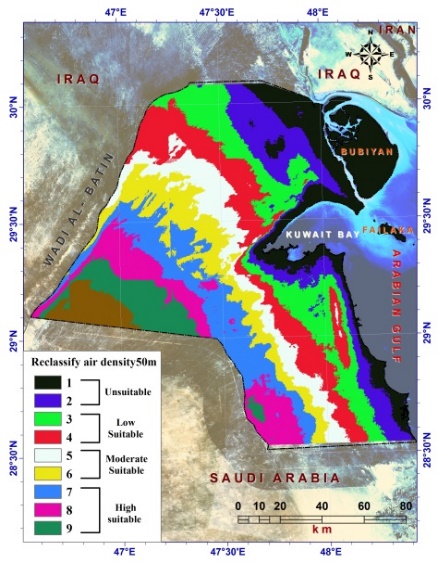

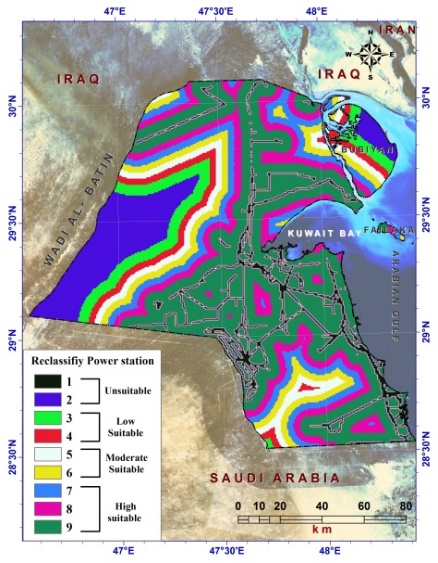


**a**

**b**

**c**

**d**


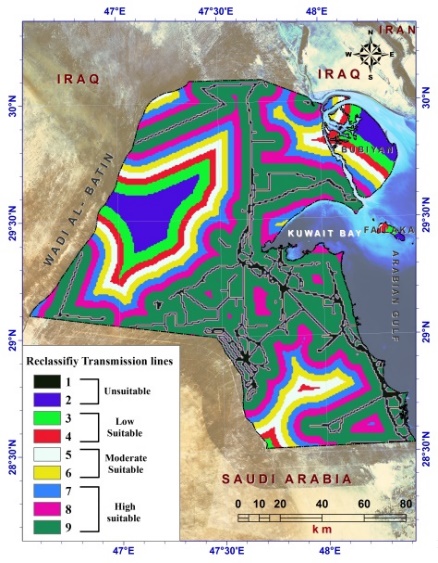


**e**

**Appendix 4: Reclassification technical criteria maps of the wind farms. (a) Reclassify Average Wind 50 m; (b) Reclassify Power Density 50 m; (c) Reclassify Air Density 50 m; (d) Reclassify Power Station; and (e) Reclassify Transmission Lines.**

Source: based on spatial analysis using a program Arc GIS V, 10.3.


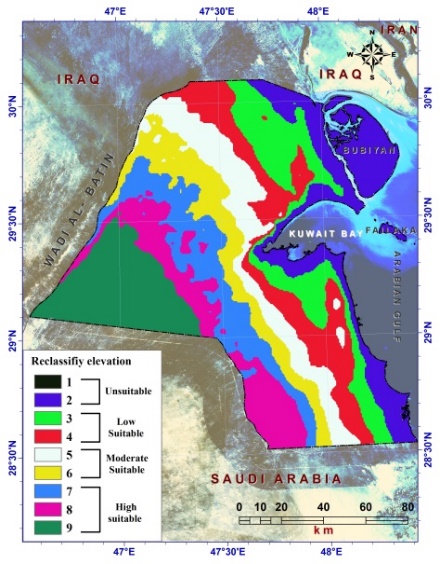

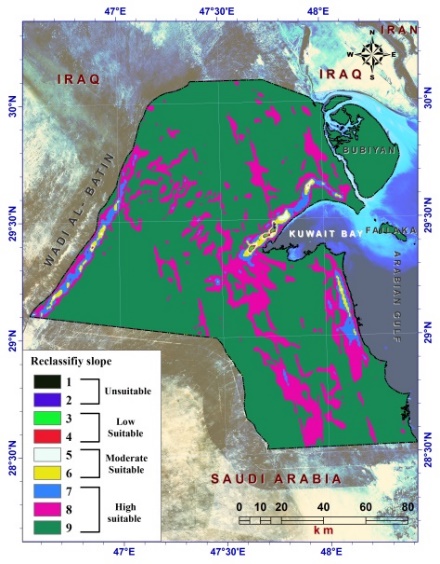


**a**

**b**


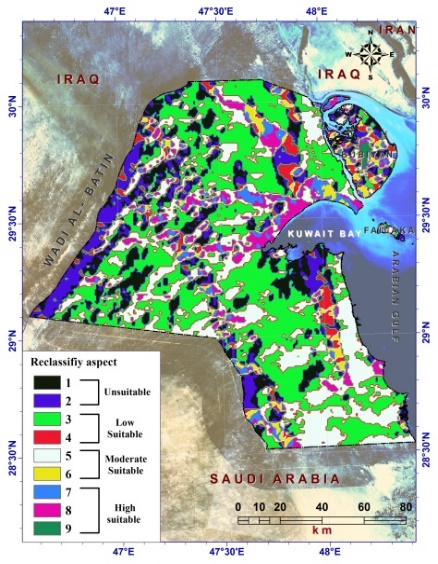


**c**


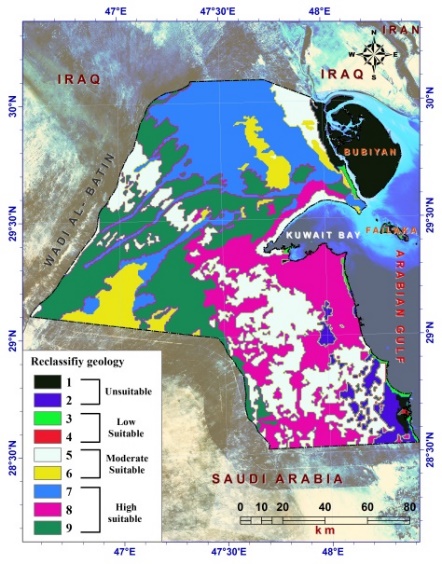


**d**


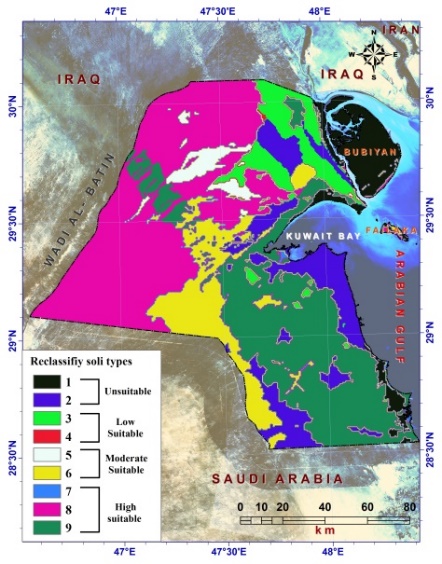


**e**

**Appendix 5: Reclassification topographic criteria maps of the wind farms. (a) Reclassify Elevation (m); (b) Reclassify Slope (%); (c) Reclassify Aspect; (d) Reclassify Geology; and (e) Reclassify Soil.**

Source: based on spatial analysis using a program Arc GIS V, 10.3.


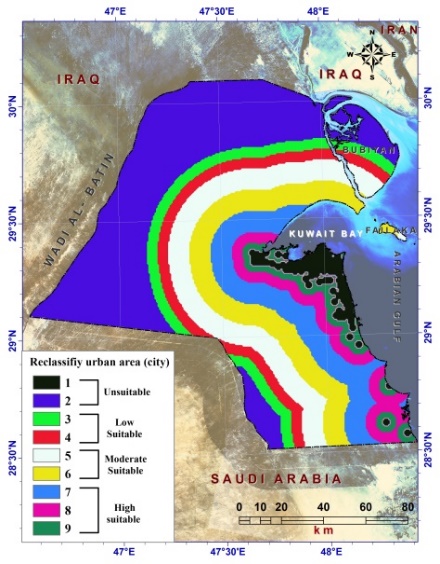

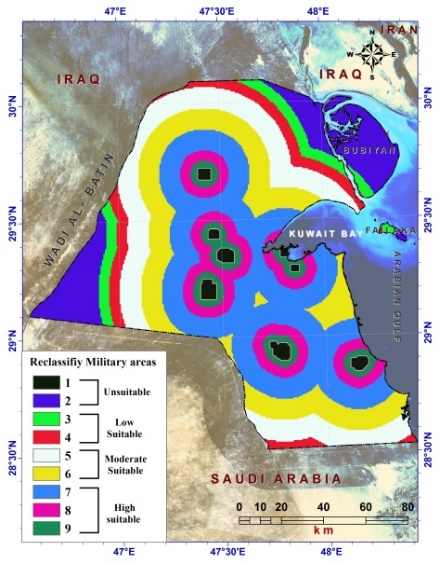


**a**

**b**


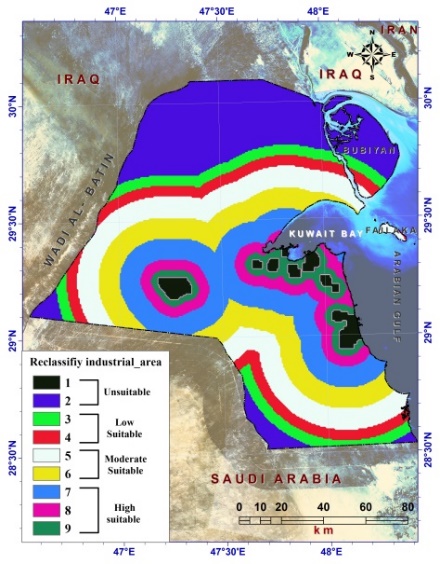

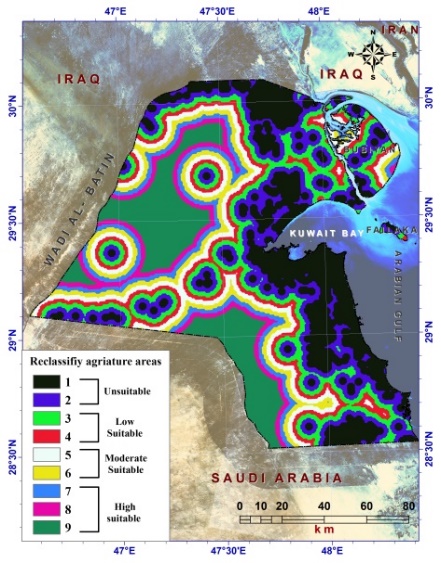

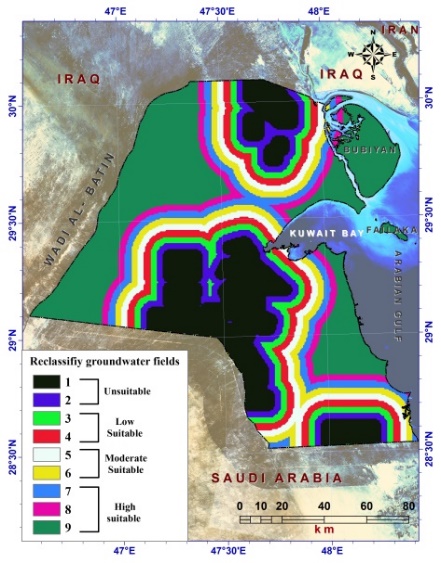

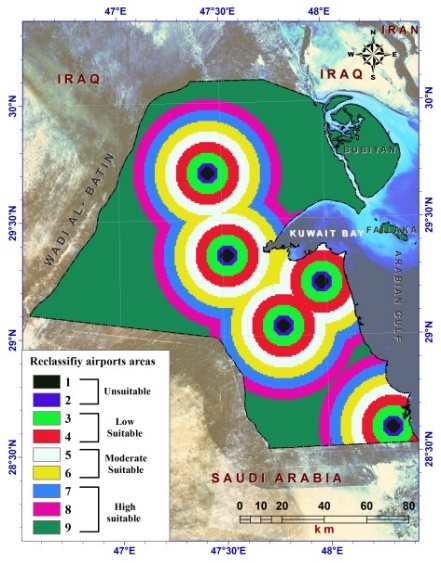


**c**

**d**

**ee**

**f**


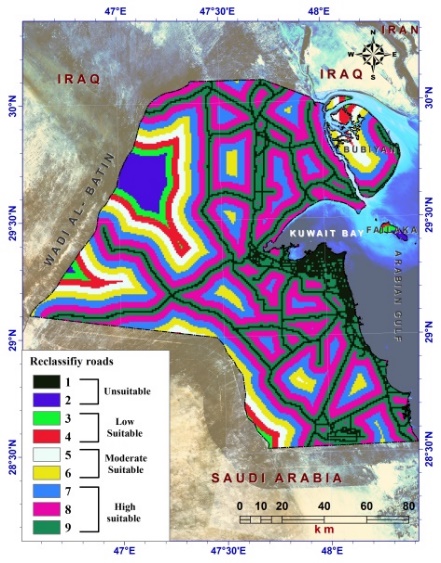

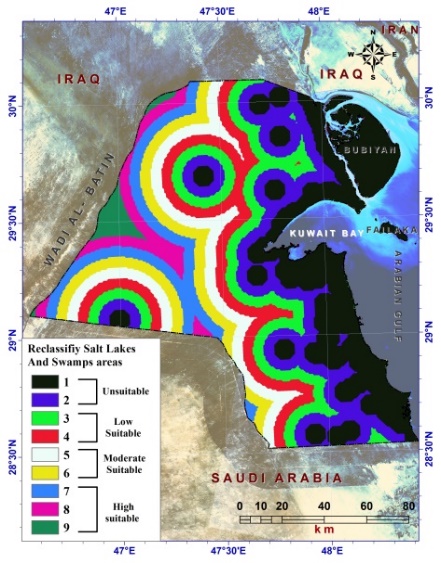

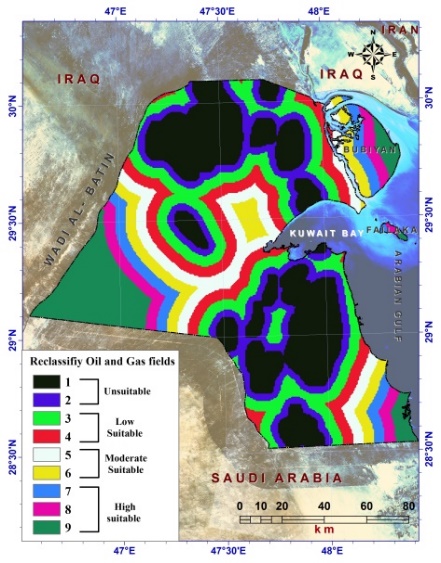


**g**

**h**

**j**

**Appendix 6: Reclassification social and economic criteria maps of the wind farms. (a) Reclassify urban area; (b) Reclassify military areas; (c) Reclassify industrial area; (d) Reclassify agriculture areas; (e) Reclassify groundwater fields; (f) Reclassify airports areas; (g) Reclassify roads; (h) Reclassify salt lakes and swamps; and (j) Reclassify oil and gas fields.**

Source: based onspatial analysis using a program Arc GIS V, 10.3.


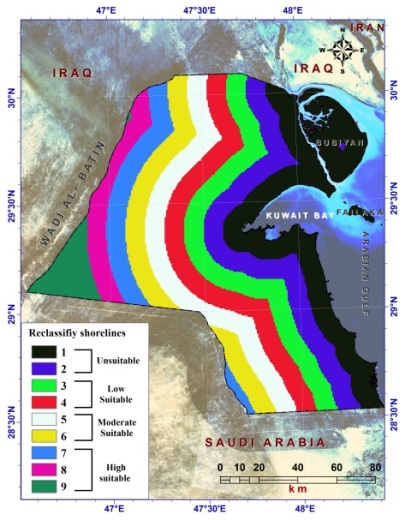

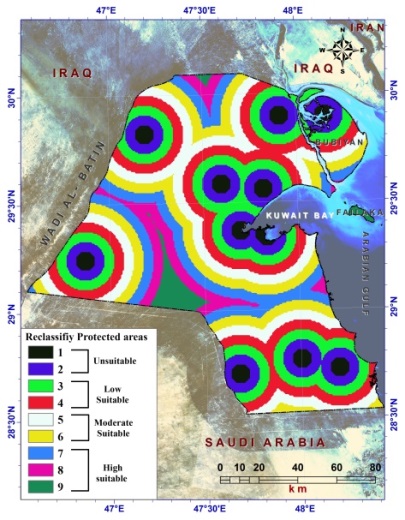

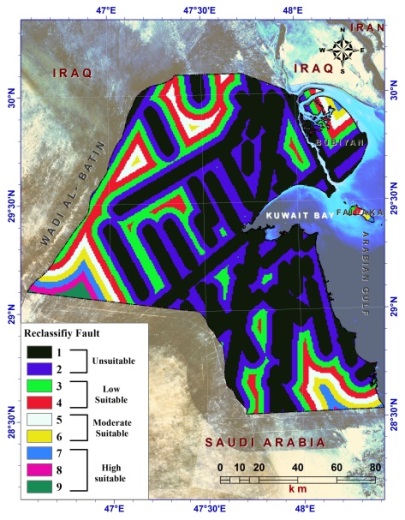

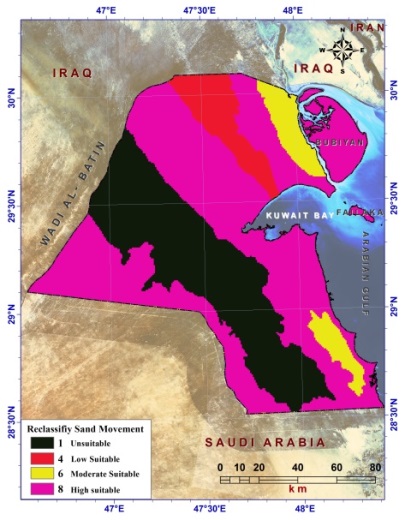


**a**

**bb**

**c**

**d**


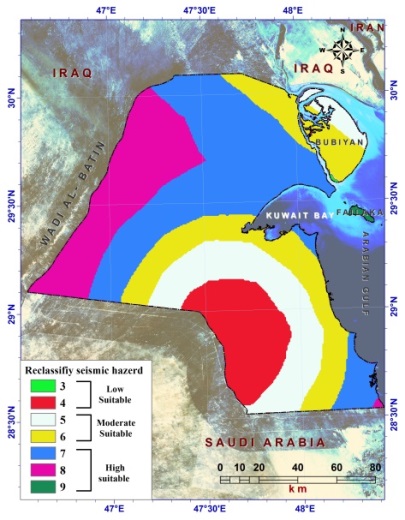

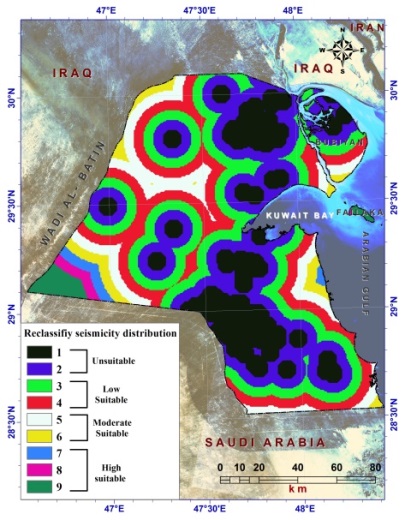

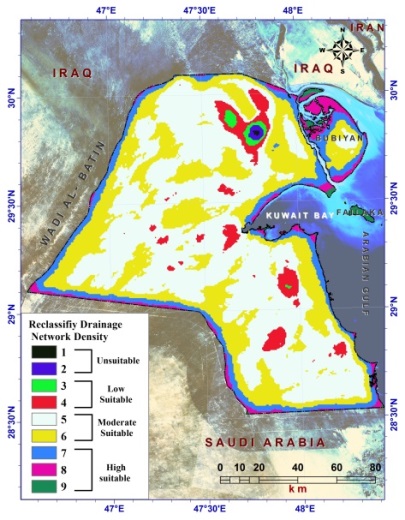


**e**

**f**

**g**

**Appendix 7: Reclassification environmental criteria maps of the wind farms. (a) Reclassify shoreline; (b) Reclassify protected areas; (c) Reclassify fault density; (d) Reclassify sand movement; (e) Reclassify seismic hazard; (f) Reclassify seismicity distribution; and (g) Reclassify drainage network density.**

Source: based on spatial analysis using a program Arc GIS V, 10.3.
